# Fungal Endophyte Comprehensively Orchestrates Nodulation and Nitrogen Utilization of Legume Crop (*Arachis hypogaea* L.)

**DOI:** 10.3390/jof12010065

**Published:** 2026-01-13

**Authors:** Xing-Guang Xie, Hui-Jun Jiang, Kai Sun, Yuan-Yuan Zhao, Xiao-Gang Li, Ting Han, Yan Chen, Chuan-Chao Dai

**Affiliations:** 1 Jiangsu Key Laboratory for Pathogens and Ecosystems, Jiangsu Engineering and Technology and Research Center for Industrialization of Microbial Resources, College of Life Sciences, Nanjing Normal University, Nanjing 210023, China; xgxie@smmu.edu.cn (X.-G.X.); jianghuijun1998@163.com (H.-J.J.); sunkainnu@sina.cn (K.S.); 17302591970@163.com (Y.-Y.Z.); 2 Department of Pharmacognosy, School of Pharmacy, Naval Medical University, Shanghai 200433, China; hanting@smmu.edu.cn; 3 Co-Innovation Center for Sustainable Forestry in Southern China, College of Biology and the Environment, Nanjing Forestry University, Nanjing 210037, China; xgli@njfu.edu.cn; 4 State Key Laboratory of Soil and Sustainable Agriculture, Institute of Soil Science, Chinese Academy of Sciences, Nanjing 210008, China

**Keywords:** fungal endophyte, legume crop, nodulation and N_2_ fixation, N utilization, N transformation related microflora, continuous cropping

## Abstract

(1) Background: Improving nitrogen use efficiency in peanuts is essential for achieving a high yield with reduced nitrogen fertilizer input. This study investigates the role of the fungal endophyte *Phomopsis liquidambaris* in regulating nitrogen utilization throughout the entire growth cycle of peanuts. (2) Methods: Field pot experiments and a two-year plot trial were conducted. The effects of *Ph. liquidambaris* colonization on the rhizosphere microbial community, soil nitrogen forms, and peanut physiology were analyzed. (3) Results: Colonization by *Ph. liquidambaris* significantly suppressed the abundance of ammonia-oxidizing archaea (AOA) and bacteria (AOB) in the rhizosphere at the seedling stage. This led to a transient decrease in nitrate and an increase in ammonium availability, which enhanced nodulation-related physiological responses. Concurrently, the peanut-specific rhizobium *Bradyrhizobium* sp. was enriched in the rhizosphere, and the root exudates induced by the fungus further stimulated nodulation activity. These early-stage effects promoted the establishment of peanut–*Bradyrhizobium* symbiosis. During the mid-to-late growth stages, the fungus positively reshaped the composition of key functional microbial groups (including diazotrophs, AOA, and AOB), thereby increasing rhizosphere nitrogen availability. (4) Conclusions: Under low nitrogen fertilization, inoculation with *Ph. liquidambaris* maintained yield stability in long-term monocropped peanuts by enhancing early nodulation and late-stage rhizosphere nitrogen availability. This study provides a promising microbe-based strategy to support sustainable legume production with reduced nitrogen fertilizer application.

## 1. Introduction

Nitrogen (N) is an essential nutrient required for agricultural productivity, with over half of the global population relying on food produced using synthetic N fertilizers [1]. Grain legumes represent a vital component of world food security and nutrition [2] and are capable of forming symbiotic relationships with rhizobia and developing root nodules that fix atmospheric N, thereby reducing dependence on synthetic N inputs [3]. However, growing population pressure and limited arable land have led to the widespread long-term monocropping of legumes—particularly in developing countries—often without rotation [2,4]. Beyond other well-documented constraints, long-term continuous cropping severely impairs legume nodulation and N_2_ fixation [4,5,6]. This fact has forced farmers to apply a large amount of exogenous N fertilizer to maintain high yields of monocropped grain legumes [7]. However, excessive N application not only threatens environmental sustainability but also further suppresses the nodulation and N_2_ fixation of grain legumes, creating a detrimental feedback loop that undermines the sustainability of legume-based systems [8]. Thus, improving the nodulation and N utilization of grain legumes in long-term continuous cropping fields is an issue that needs to be solved urgently.

The establishment of symbiotic interactions between legumes and rhizobia involves complex molecular dialogue, generally comprising two critical phases: activation of rhizobia and transduction of symbiotic signal in host plants [3,9]. Initially, legume roots release exudates into the rhizosphere that recruit compatible rhizobia partners. Specific components of these exudates—such as phenolics and flavonoids compounds—activate key nodulation-related processes in rhizobia, including chemotaxis, biofilm formation, and nodulation gene expression, thereby inducing the production of the Nod factor (NFs) lipochitooligosaccharide [9,10,11]. Upon perception of NFs by the host plant, a series of intracellular processes are triggered, leading to the expression of symbiotic genes and the initiation of nodule primordia. This stage involves multiple signaling components—such as phytohormones, ions, peroxides, and gas signaling molecules—that collectively regulate legume–rhizobia symbiosis [9,11]. Therefore, the optimal nodulation-related physiologies of both the host plants and the rhizosphere rhizobia are crucial for forming the active nodules of legumes.

Although legumes can form nodules and fix atmospheric nitrogen, this natural nitrogen supply often fails to meet the full N demand of the host plants throughout the entire growth cycle in long-term farming systems. Therefore, soil-native nitrogen or supplemental N fertilizers are often necessary to support high yields in grain legumes [8]. Especially when the crops grow to podding and maturing periods, if the overall N supply does not meet requirements, the crops will remobilize N accumulated in leaves to the grain, which diminishes the photosynthetic capacity of the canopy and thus limits yield potential [8], indicating the significance of increasing N acquisition at the mid–late stage of grain legumes. Therefore, optimizing the balance between nodulation and rhizosphere N availability is essential for maintaining legume crop growth and yield under low-N-input conditions. The rhizosphere’s microecological environment—particularly its functional microbial communities and nutrient availability—plays a central role in regulating key plant processes [6,12,13]. For instance, the abundance and diversity of rhizosphere N-fixing rhizobia directly influence nodulation and N_2_ fixation efficiency [10]. Moreover, ammonia-oxidizing archaea (AOA) and bacteria (AOB) govern nitrogen transformations between ammonium (NH_4_^+^-N) and nitrate (NO_3_^−^-N), which not only shape nitrogen forms available for plant uptake but also indirectly modulate legume–rhizobia symbiosis [9,14,15,16]. Therefore, improving the rhizosphere microecological environment holds promise for enhancing N utilization across the growth stages of long-term monocropped grain legumes. However, associating this with promoting nodulation and improving N availability still faces great challenges.

China is a major agricultural producer, yet its domestic legume output remains insufficient to meet industrial demand. Currently, China is the world largest bean importer, and it imported more than 60% of global exported soybeans (93.5 million metric tons) in 2016 [17,18]. Against this backdrop, enhancing the productivity of key domestic legumes—particularly peanut (*Arachis hypogaea* L.), a globally significant oil and cash crop—has gained renewed urgency [10,19]. China is the leading country in peanut production, contributing roughly 40% of the global total [10], underscoring the crop’s strategic importance for ensuring food security amid international trade uncertainties. Our laboratory previously isolated an endophytic fungus, *Phomopsis liquidambaris* C.Q.Chang, Z.D.Jiang & P.K.Chi (Ascomycota, Sordariomycetes, Diaporthaceae), from *Bischofia polycarpa* (H.Lév.) Airy Shaw (Phyllanthaceae). Subsequent research has shown that this endophytic fungus establishes a stable symbiotic relationship with peanut, enhancing nodulation and N_2_ fixation [5,20,21]. Multiple mechanisms underlying these effects have been elucidated under controlled conditions, including modulation of phytohormones [22], activation of nodulation signaling [20], increased leaf CO_2_ fixation [5], improvement of rhizosphere microbiota and facilitation of rhizobial transfer via mycelial networks [23]. Notably, although *Ph. liquidambaris* colonizes peanuts only until the flowering stage, it induces a sustained increase in nitrogen accumulation throughout the growth cycle—a legacy effect indicating profound physiological and ecological impacts [21]. However, the strategies and mechanisms by which this fungal endophyte regulates whole-plant nitrogen utilization in field-grown monocropped peanuts remain largely unexplored.

Therefore, the ultimate objective of this work was to reveal how fungal endophyte colonization comprehensively orchestrates N utilization in long-term continuously cropped legume peanuts throughout the whole growth period. The main research directions were to analyze the potential mechanisms between the improvement of the rhizosphere’s microecological environment and the promotion of nodulation in the early stage and the improvement of N availability in the mid-late stage of peanuts. This study is the first to create an overall plan for fungal endophytes in host N utilization from the perspective of the whole growth stage, which might be helpful for reducing N fertilizer inputs and maintaining yield in the long-term monocropped field of grain legumes. This broadens the ecological functions of endophytic fungi.

## 2. Materials and Methods

### 2.1. Plant and Functional Microbial Strain Supply

For the endophytic fungus *Ph. liquidambaris*, the strain was previously isolated from the inner bark of the stem of *B. polycarpa* and stored at 4 °C on potato dextrose agar medium [20]. The fungus was activated at 28 °C in potato dextrose broth medium for 48 h at 180 rpm in an orbital shaker. Fungal mycelia were collected by filtering, and the preparation of the fungal inoculum was conducted in accordance with our previous study [20].

For *Bradyrhizobium* strains, the *Bradyrhizobium yuanmingense* Yao et al., 2002 (Bacteria, Proteobacteria, Xanthobacteraceae) strain (CCBAU21353) was obtained from the Culture Collection of Beijing Agricultural University. In addition, three native *Bradyrhizobium* strains of peanuts, in this study, were isolated from the surface-sterilized root nodules of peanuts using standard isolation techniques [24] and purified using the streaking method. Pure strains were identified as *Bradyrhizobium japonicum* C (B-1), *Bradyrhizobium* sp. (B-2), and *Bradyrhizobium liaoningense* C (B-3), respectively, and their nodulation capabilities for peanuts were evaluated and are presented in Figure S6. All four *Bradyrhizobium* strains were stored in yeast extract mannitol agar medium (YEMA) slant and, respectively, activated using YEM medium before use.

The legume peanut (*A. hypogaea*, cultivar Ganhua-5) was used to study microbial–plant interactions. Peanut seedlings were artificial colonized with the endophytic fungus *Ph. liquidambaris*, and the fungal colonization status in roots was further confirmed under a fluorescence microscope. Seedlings colonized by the fungal endophyte *Ph. liquidambaris* were marked as PL+, and those without *Ph. liquidambaris* colonization were market as PL-. After raising seedlings, PL+ and PL- seedlings at similar growth stages were transplanted for the following experimental studies, respectively. 

### 2.2. Experimental Soil Types

Our previous studies have demonstrated that the fungal endophyte *Ph. liquidambaris* colonization could effectively increase host peanut nodulation and N accumulation in the laboratory [5,20,22,23]. To confirm whether such microbial promotion is universal in long-term continuous cropping systems and further elucidate the underlying mechanisms, we selected two soil types, which were widely distributed in temperate and subtropical regions, representing intensive row peanut agricultural systems in China.

(1) Red soil: soil was collected (0–20 cm surface layer) from a long-standing (over 10 years) peanut field at the Ecological Experimental Station of Red Soil at the Chinese Academy of Sciences (Jiangxi province, China, 28°13′ N, 116°55′ E). The soil was a typical acidic loamy clay classified as Ferralic Cambisol [25]. The basic properties of acidic red soil were described as follows: organic matter, 9.66 mg kg^−1^; total N, 0.65 g kg^−1^; total P, 0.46 g kg^−1^; total K, 12.65 g kg^−1^; NH_4_^+^-N, 1.25 mg kg^−1^; NO_3_^−^-N, 6.32 mg kg^−1^; available P, 13.28 mg kg^−1^; available K, 106.04 mg kg^−1^; pH 5.6 (soil to water ratio: 1:2.5). The collected red soil was transferred to Nanjing Normal University Botanical Garden for field pot experiments 1 and 3.

(2) Yellow-brown soil: soil was taken from a 10-year-old peanut monocropping field at Nanjing Normal University Botanical Garden (Jiangsu province, China, 31°14′ N, 118°22′ E). This soil is a typical yellow-brown soil classified as Ferri-Udic Argosol [25]. The soil’s basic properties are as follows: organic matter, 16.38 mg kg^−1^; total N, 0.85 g kg^−1^; total P, 0.62 g kg^−1^; total K 9.58 g kg^−1^; NH_4_^+^-N, 2.36 mg kg^−1^; NO_3_^−^-N, 9.06 mg kg^−1^; available P, 12.15 mg kg^−1^; available K, 108.63 mg kg^−1^; pH 7.2 (soil to water ratio: 1:2.5). We used this soil for field plot experiment 2.

### 2.3. Field Experiment 1 Design

To begin with, 180 whole peanut seedlings (18 replicates × 5 periods × 2 treatments) at similar growth stages were, respectively, transplanted into a pot containing 12 kg of fresh red soil. In each pot (28 cm × 23 cm, diameter × height) triplicate seedlings were transplanted, meaning a total of 60 pots were prepared. Pots containing fungal endophyte *P. liquidambaris*-colonized seedlings were marked as PL+ treatment (90 seedlings). PL- seedlings were plated in an identical manner and marked as controls (90 seedlings). Five sampling timepoints were used during peanut growth (including nodule initiation (7 days), seedling (15 days), flowering (45 days), podding (75 days), and maturing (105 days) periods), and 18 plants were sampled from each treatment group during each period. Field pots were randomly placed in the Nanjing Normal University Botanical Garden and were weeded and watered by hand each week. Peanut plants were collected and used to determine peanut nodulation-related parameters, physiological responses, and plant tissue N content and biomass. In addition, 18 rhizosphere soil samples that tightly adhered to the peanut roots were collected and stored at −80 °C for soil property and microbial molecular determination. Detailed detection indicators and analysis steps are described below.

### 2.4. Field Experiment 2 Design

To confirm the broader availability and stability of the effects of *Ph. liquidambaris* stimulation on peanut nodulation and N utilization, we further performed a consecutive 2-year-long field plot experiment (2017–2018) at Nanjing Normal University Botanical Garden. Each treatment, with triplicate randomized blocks, was carried out in a 4 m × 3 m (length × width) plot. The plots were separated by a 1 m (wide) buffer zone. The plots were cultivated with peanuts from 10–20 April to 15–25 August every year, starting in 2006. Each plot was fertilized with 180 g urea (medium N), 900 g calcium magnesium phosphate, and 67.5 g potassium chlorate. During 2017–2018, 10 days after fertilization, fungal endophyte *Ph. Liquidambaris*-colonized and noncolonized peanut seedlings were transplanted into plots and marked as PL+ (300 seedlings) and PL- treatments (300 seedlings), respectively. Each hole contained two seedlings. Each plot contained 10 rows of peanuts (with an inner-row spacing of 0.4 m and an inter-plant spacing of 0.2 m). Consistent with the sampling in field experiment 1, plants and rhizosphere soil samples were collected during nodule initiation, seedling, flowering, podding, and maturing periods for the corresponding experimental detection method mentioned above.

### 2.5. Exogenous N Gradient Concentration Experiment

To explore whether the level and form of soil inorganic N were responsible for affecting peanut–rhizobia symbiosis and nodulation, peanut exogenous inoculation of *Bradyrhizobium* at 4 NH_4_^+^-N and NO_3_^−^-N concentrations was conducted in pots. Briefly, peanut seedlings were firstly surface-inoculated with the *B. yuanmingense* strain by dipping seedling roots into the strain suspension (OD_600_ 0.8), and then they were, respectively, transplanted into plastic pots (20 cm × 18 cm, diameter × depth) containing sterilized vermiculite. Then, 400 mL of basic nutrient solution containing 0, 0.1, 0.5, and 2.5 mM NH_4_^+^-N [(NH_4_)_2_SO_4_] or NO_3_^−^-N (KNO_3_) (pH = 6.0), respectively, was sprayed onto the seedlings every two days [26]. To eliminate the influence of other ions in the experiment, potassium sulfate (K_2_SO_4_) was used to balance the concentration of K^+^ and SO_4_^2−^ in all treatments. All pots were cultivated and randomly distributed in a growth chamber at 28 °C with 16 h of light and at 25 °C with 8 h of darkness, with 70% relative humidity. Eighteen peanut plants from each treatment group were collected, respectively, for the determination of nodulation-related physiological response (7 days) and nodulation parameter (45 days) analyses.

### 2.6. Field Experiment 3 Design

To clarify the practical potential of the fungal endophyte *Ph. liquidambaris* to maintain long-term monocropped peanut yield under low-N-fertilizer conditions, exogenous co-inoculation with peanut-native *Bradyrhizobium* in the field was performed. Three activated native *Bradyrhizobium* strains (B-1, B-2 and B-3) isolated from peanut root nodules (field experiment 1) were washed and diluted at OD600 = 0.8, respectively. Then, 3 individual bacterial suspensions were mixed at an equal volume and used as *Bradyrhizobium* mixed inoculant (BMI). PL+ and PL- seedlings were first surface-inoculated with rhizobium by dipping their roots into fresh BMI, and then they were marked as PL+BMI and PL-BMI, respectively. PL+ seedlings with and PL- seedlings without BMI inoculation were processed in an identical manner. Plant treatments were transplanted into pots, where soil type was consistent with field experiment 1 mentioned above. Considering local N fertilizer usage (300 kg ha^−1^ urea) during peanut cultivation in southern China, we set up two N concentrations by adding urea: (1) low N, 0.75 g of N per pot, and (2) normal N, 2.25 g of N per pot (12 kg soil per pot). Besides N, 5 g of calcium magnesium phosphate and 4 g of potassium chlorate were applied as basal nutrition. In total, 144 pots (4 plant treatments × 2 N levels × 6 replicates × 3 periods) were prepared, with each pot containing 3 seedling individuals, resulting in a total of 432 plants. Pots were randomly arranged in the Nanjing Normal University Botanical Garden and regularly watered and weeded by hand throughout the entire growing season. After 7, 45, and 90 days of peanut growth (nodule initiation, flowering, and mid–late stages, respectively), plants and rhizosphere soil samples were collected for the corresponding detection analysis described in field experiment 1. In addition, peanut yield was also evaluated after the harvest.

### 2.7. Root Exudate Collection and Experiment Design

We tracked the dynamic changes in root exudates throughout the development period of peanuts under the field conditions. Plant samples from field experiment 1 collected during the seedling, flowering, podding, and maturing periods were, respectively, used to collect root exudates first. Briefly, the plants were washed with sterile water and placed in hydroponic culture medium away from light for 24 h; then, the hydroponic liquid was freeze-dried to collect the root exudates. The concentration of total C, total N, soluble sugar, amino acids, organic acids, phenolics, and flavonoids in the root exudates were monitored as previously mentioned [27]. Briefly, total C and total N were monitored by an Nc2500 elemental analyzer and a Kjeldahl nitrogen analyzer, while soluble sugar, amino acids, organic acids, phenolics, and flavonoids were monitored by high-performance liquid chromatography. As the organic acid, phenolic, and flavonoid compounds induced by fungal endophyte *Ph. liquidambaris* colonization play important roles in peanut–*Bradyrhizobium* symbiotic interactions, we further investigated the effects of these specific root exudate components on nodulation-related biological activities (chemotaxis ability was tested using the capillary tube method, biofilm formation was tested by crystal violet staining, and nodulation-related gene (nod C) expression was tested by qRT-PCR) for 3 peanut-native *Bradyrhizobium* strands (mentioned above). In this section, the detailed experimental procedures, such as root exudate collection, detection, and identification, specific root exudate component treatment, and the detection of *Bradyrhizobium* chemotaxis, biofilm formation, and nod C gene expression, are consistent with our previous reports [27].

### 2.8. Soil Properties and Potential Nitrification Rate Determination

We measured 12 soil properties, including soil pH, total organic carbon (TOC), dissolved organic C (DOC), dissolved organic N (DON), total nitrogen (TN), total phosphorus (TP), total potassium (TK), available phosphorus (AP), available potassium (AK), NH_4_^+^-N, and NO_3_^−^-N. The measurement methods followed those used in a recent study [28]. Briefly, soil pH, TOC, DOC, TN, TP, TK, AP, AK, NH_4_^+^-N, and NO_3_^−^-N were tested using the electrometric method, an Nc2500 elemental analyzer, a Kjeldahl nitrogen analyzer, molybdenum-blue colorimetry, and a flame spectrophotometer. Soil potential nitrification rate (PNR) was measured using the chlorate inhibition method. Briefly, 5.0 g of a fresh soil sample was added to a 50 mL centrifuge tube containing 20 mL of phosphate-buffered solution with a final concentration of 1 mM (NH_4_)_2_SO_4_. Potassium chlorate with a final concentration of 10 mM was added to inhibit nitrite oxidation. The soil suspension was then incubated in a dark incubator at 25 °C for 24 h, after which nitrite was extracted with 5 mL of 2 M KCl and measured by a spectrophotometer at 540 nm with N-(1-naphthyl) ethylenediamine dihydrochloride.

### 2.9. Molecular Determination of Soil N Transformation-Related Microbial Abundance

Soil DNA was extracted from 0.5 g of lyophilized samples using a FastDNA spin kit (MP Biomedicals, Santa Ana, CA, USA) according to the manufacturer’s protocols. The concentration and purity of the extracted DNA were assessed by a UV spectrophotometer (NanoDrop 2000, Thermo Scientific, Waltham, MA, USA). The abundances of diazotrophs (nifH gene), ammonia-oxidizing archaea (AOA, arch-amoA gene), and bacteria (AOB, amoA gene) were determined by quantitative real-time PCR (qPCR) using the primer pairs nifH-F/nifH-R [29], CrenamoA23f/CrenamoA616r [30] and amoA-1F/amoA-2R [31], respectively. The detailed operation steps of qPCR were consistent with our previous studies [21]. The abundances of the diazotrophs, AOA, and AOB were expressed as the number of corresponding gene copies per gram of dry soil, respectively.

### 2.10. Terminal Restriction Fragment Length Polymorphism (T-RFLP) Analysis

In this study, the T-RFLP analysis was used to investigate the community structures of diazotrophs, AOA, and AOB in the different experimental treatment groups. PCR amplifications were performed using the primers nifH-F/nifH-R, CrenamoA23f/CrenamoA616r, and amoA-1F/amoA-2R, as mentioned above, with the forward primers nifH-F, CrenamoA23f, and amoA-1F labeled with 6-carboxyfluorescein (FAM), respectively. The PCR reaction conditions were the same as those used for the qPCR assays. After amplification, PCR products were purified using the PCR Cleanup Kit (Axygen Biosciences, Union City, CA, USA) and then confirmed by 1.0% agarose gel electrophoresis. The concentrations of purified PCR products were fluorometrically quantified with the Qubit dsDNA HS Assay Kit (Invitrogen, Carlsbad, CA, USA) according to the manufacturer’s protocols. Restriction enzyme digestions were performed in a 10 μL mixture containing approximately 200 ng of purified PCR products, 0.1 μL of BSA (bovine serum albumin), 1 μL of 10 × NE Buffer, and 5 U of the restriction enzymes HaeIII for diazotrophs and MboI for AOA or AOB (Bio-Labs, Sydney, NSW, Australia). Digestions were performed at 37 °C for 3 h and then denatured at 95 °C for 10 min to deactivate the restriction enzymes. The digested products were size-separated in an ABI 3730xl DNA Analyzer (Applied Biosystems, Waltham, MA, USA).

### 2.11. Cloning and Sequencing Analysis of T-RFs

To identify the T-RFs, the clone libraries of the nifH, arch-amoA, and amoA genes were constructed using the same primers as T-RFLP but without 6-FAM labeling. The PCR conditions and product purification were consistent with T-RFLP analysis. To identify as many T-RFs detected in the T-RFLP as possible, we individually mixed the PCR products of the nifH, arch-amoA, and amoA genes from the PL+ and PL- soil samples to, respectively, construct mixed diazotrophs, AOA, and AOB clone libraries. The purified PCR products were cloned into the pEASY-T1 vector and then transformed into Escherichia coli DH5α cells, following the manufacturer’s instructions (TransGen Biotech, Beijing, China). Approximately 150 positive clones per library were randomly selected and then sequenced using an ABI 3730 DNA sequencer (Applied Biosystems). The obtained sequences were subjected to homology analysis using the software DNAMAN, version 6.0.3.48 (Lynnon Biosoft, San Ramon, CA, USA). Sequences displaying more than 97% similarity with each other were classified into the same operational taxonomic units (OTUs), and only one representative sequence of each OTU was compared with the sequences in the Basic Local Alignment Search Tool (BLAST, https://www.ncbi.nlm.nih.gov/ accessed on 1 May 2018)/National Center for Biotechnology Information (NCBI) database.

### 2.12. Detection of Peanut–Bradyrhizobium symbiosis-Related Gene Expression

The peanut roots’ total RNA was extracted from 0.2 g of tissue using TRIzol reagent (Invitrogen, Carlsbad, CA, USA), according to the manufacturer’s recommendations. The extracted RNA was purified with RNase-free DNaseI (Invitrogen) and determined by spectrophotometry (NanoDrop 2000, Thermo Scientific, USA). RNA was reverse-transcribed into single-stranded cDNA using a PrimeScript RT Reagent Kit (Takara, Dalian, China). qPCR was performed on StepOne Real-time PCR systems (Applied Biosystems) using a One-step RT-PCR kit with SYBR Green I fluorescent dye (Vazyme Biotech, Nanjing, China). Peanut Actin and Ubiquitin were used as internal controls [20], and the specific primer pairs used for quantitative measurement of the transcription levels of SymRK, CCaMK, NIN, and ENOD40, respectively, were consistent with our previous reports. The reaction mixture (20 µL) contained 10 µL of SYBR^®^ Green Master Mix (Vazyme), 0.5 µM of forward and reverse primers, and 0.2 ng of cDNA template. The amplification programs were performed with the following parameters: 94 °C for 1 min, followed by 45 cycles of 94 °C for 15 s, 60 °C for 45 s, and 72 °C for 30 s. The relative expressions of target genes were normalized to the levels of the two reference genes and calculated using the 2^−ΔΔCt^ method.

### 2.13. Peanut Physical and Nodule Parameter Measurements

Nodule number and nodule dry weight (DW) were measured, and other nodulation-specific parameters, such as nodule number g^−1^ root DW and nodule DW g^−1^ root DW, were evaluated according to a previous study [15]. Leghemoglobin content was analyzed according to the methods of Medeiros-Silva et al. described in [32], and nitrogenase activity was determined using the acetylene reduction assay, as described by Quilliam et al. [33]. The biomass of peanut nodules, shoots, roots, and pods was calculated after heating at 80 °C to constant weight. The N concentrations in the shoots, roots and pods were determined using the Kjeldahl method.

### 2.14. Root Symbiotic Signaling Molecule Detection

Phytohormones, ions, peroxides, and gas signaling molecules in plants have been reported to be important in mediating legume–rhizobia symbiosis and nodules [3,9,11,20,22]. Here, we chose 6 plant signals [including auxin, cytokinin, ethylene, calcium ion (Ca^2+^), hydrogen peroxide (H_2_O_2_), and nitric oxide (NO)] to track peanut–*Bradyrhizobium* interactions and nodule formulation. The details of the high-performance liquid chromatography methods were described in our previous reports [20,22].

### 2.15. Statistical Analyses

Statistical analyses were performed using SPSS 13.0 (SPSS Inc., Chicago, IL, USA), and all data were represented by the average of three biological replicates and their standard deviations (±SD). The normal distribution of data and the homogeneity of variances were checked by Shapiro–Wilk’s test and Levene’s test, respectively. If needed, the original values were log-transformed for further analysis. When an analysis consisted of only a control and an experimental group, an independent t test was performed, and when three or more groups were compared, a one-way analysis of variance (ANOVA) was performed, followed by Tukey’s multiple comparison test. In addition, when the time factor was considered in the experiments, the data significance was analyzed by using ANOVA for repeated measures and Bonferroni’s post hoc test, and *p* < 0.05 was considered significant. The relative abundance of individual terminal restriction fragments (T-RFs) was calculated as the percentage of the total peak area in the T-RFLP profile using GeneMarker V2.2 (ABI, Los Angeles, CA, USA). Only those T-RFs with a relative abundance > 1% and fragment lengths in the range of 50–500 bp for diazotroph, 50–600 bp for AOA, and 50–500 bp for AOB were considered in the subsequent data analyses. Diversity indices, including the Shannon (H) and evenness index (E), were calculated according to the formulae described in previous reports [34]. The relationships between rhizosphere diazotroph, AOA, and AOB community structures (based on the relative abundance of T-RFs) and soil properties were investigated by using redundancy analysis (RDA) after checking the length of the gradient of the T-RFLP dataset through detrended correspondence analysis (DCA) in CANOCO software, version 4.5 (Biometry, Wageningen, The Netherlands). A Monte Carlo permutation test was performed to test the significance of the first and all canonical axes in the RDA.

## 3. Results

### 3.1. Endophytic Fungus Phomopsis liquidambaris Colonization Improves Peanut Nodulation and N Resource Distribution in Host and Rhizosphere Soil

In both red soil and yellow-brown soil, field monocropped peanuts colonized with the fungal endophyte *Ph. liquidambaris* showed a higher nodule number, nodule dry weight, specific nodule parameters, and N_2_ fixation activity (Figures S1a–d and S2a–f). As a result, the N concentrations and the biomass of PL+ tissues were higher than those in PL- tissues (Figures S1i–m and S2g–k) throughout the entire growth stage over two years. In parallel, endophytic fungus *Ph. liquidambaris* colonization affected the availability of peanut rhizosphere N (Figure S3). The concentration of NH_4_^+^-N in the PL+ rhizosphere remained 23–70% higher than that of PL- throughout the entire growth period in both field experiments (Figure S3a,d). On the other hand, the NO_3_^−^-N concentration in the rhizosphere of PL- was 28–42% higher than that of PL+ at the seedling stage (Figure S3b,e). However, during the podding and maturing stages, the PL+ rhizosphere always showed a higher NO_3_^−^-N level than the PL- treatment. This was consistent with the dynamics of the rhizosphere PNR, indicating the feasibility of nitrate production from ammonium (Figure S3c,f).

### 3.2. Endophytic Fungus Phomopsis liquidambaris-Colonized Peanuts Affect the Abundances of N-Transforming Genes in the Rhizosphere

**Figure 1:**
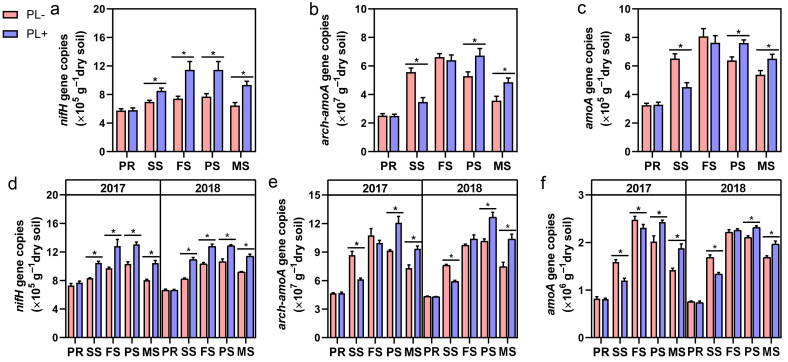
*Phomopsis liquidambaris* colonization significantly affects the abundance changes of rhizosphere N transformation-related microorganisms. Abundances of N-transforming microbial communities [*nifH* (diazotrophs), *arch-amoA* (AOA), and *amoA* (AOB) genes] in peanut rhizospheres in field experiment 1 (**a**–**c**) and field experiment 2 (**d**–**f**) throughout the entire growth stage. Data and errors are mean ± SD (n = 3), and asterisks indicate significant differences between the PL- and PL+ treatments at each growing stage (Student’s *t*-test; *p* < 0.05). Each biological replicate represents a pooled sample from at least six individual rhizosphere soil samples. PL-, noncolonized peanuts; PL+, *Ph. Liquidambaris*-colonized peanuts; PR, presowing stage; SS, seedling stage; FS, flowering stage; PS, podding stage; MS, maturing stage.

Functional genes participating in N_2_ fixation (*nifH*) and nitrification (*arch-amoA, amoA*) were used as biomarkers to determine the abundances of rhizosphere diazotrophs and ammonia oxidizers [ammonia-oxidizing archaea (AOA) and bacteria (AOB)]. In both field experiments (1 and 2), the abundances of the *nifH* gene in the PL+ rhizosphere were 21–54% higher than those in the PL- rhizosphere throughout the entire growth stage (Figure 1a,d). For N nitrification, the abundances of *arch-amoA* were significantly higher than those of *amoA* (Figure 1b,c,e,f), indicating that AOA is the dominant community for rhizosphere nitrification. Interestingly, PL+ showed a markedly lower abundance of AOA and AOB than PL- during the seedling stage, but this slowly increased to the same level as PL- at the flowering stage, and then PL+ exhibited higher abundances than PL- from the podding to maturing stages. This is consistent with the dynamics of rhizosphere NO_3_^−^-N availability.

### 3.3. Peanut Colonization with Fungal Endophyte Phomopsis liquidambaris Regulates Rhizosphere N-Transforming Microbial Community

The relative abundances of T-RFs in the T-RFLP profiles were used to determine the communities of rhizosphere diazotrophs, AOA, and AOB. For diazotrophs, in two field experiments (1 and 2), the total number of T-RF genotypes obtained through the digestion of *nifH* sequences in the PL+ rhizosphere was significantly higher than that in the PL- treatment group (Tables S1 and S4). Interestingly, in comparison with the PL- rhizosphere, it was observed that all T-RF genotypes closely related to the genus *Bradyrhizobium* showed higher relative abundance in the PL+ rhizosphere at the seedling stage of peanuts (Tables S1, S2, S4 and S5). However, as the peanut developed, the relative abundances of *Brachyrhizobium* sp. in PL+ gradually decreased, but T-RFs belonging to N-fixing bacteria significantly increased. Tables S3 and S6 show that the PL+ treatments always resulted in a relatively higher diversity of diazotrophs throughout the entire cultivation stage when compared to the PL- groups.

Consistent with the dynamics of specific N-transforming microbial abundances in the two field experiments, the number of AOA T-RF genotypes was always higher than that of AOB genotypes, as determined through the digestion of the *arch-amoA* and *amoA* sequences, respectively (Tables S1 and S4). Further analysis found that, for both the field red soil and consecutive two-year yellow-brown soil experiments, the number of T-RF genotypes representing AOA and AOB rhizospheres and their diversities in terms of T-RFLP profiles was lower in the PL+ rhizosphere than in the PL- rhizosphere at the seedling stage (Tables S1, S3, S4 and S6), which was further confirmed by observing that some specific T-RF genotypes of AOA and AOB were significantly inhibited by the PL+ treatment (Tables S2 and S5). However, after the flowering stage, the inhibitory potential of these specific T-RFs was weakened, and the PL+ rhizosphere showed higher numbers and diversities of AOA and AOB T-RF genotypes than those in the PL- treatment groups. In addition, it was also noticed that some T-RF genotypes of AOA and AOB could be sustained throughout the entire growth period of peanuts, while others were only detected in a specific growth stage in different treatment groups.

**Figure 2:**
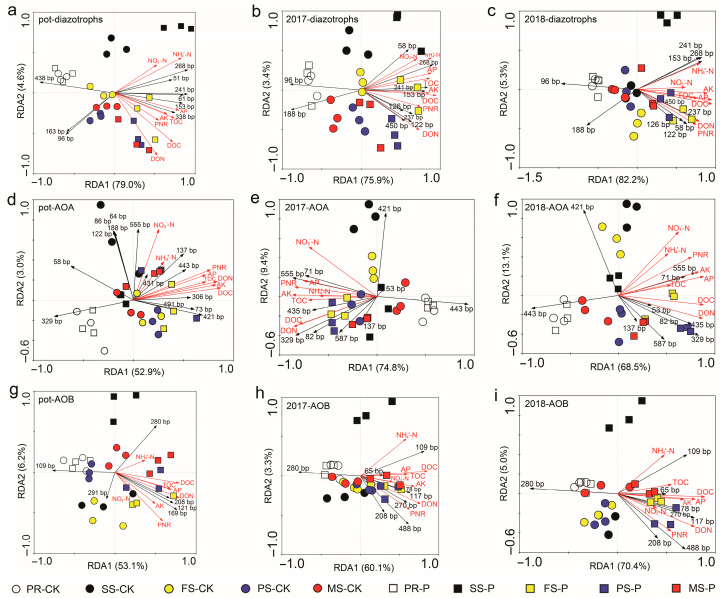
*Phomopsis liquidambaris* colonization reshapes the community structures of rhizosphere N transformation-related microorganisms. Redundancy analysis (RDA) of rhizosphere soil diazotroph (**a**–**c**), AOA (**d**–**f**), and AOB (**g**–**i**) communities generated by the T-RFLP profiles throughout the entire growth stage of peanuts in field experiment 1 and field experiment 2, respectively. The T-RFLP experiment was performed with three independent biological replicates, with each biological replicate representing a pooled sample from at least six individual rhizosphere soils. Pot-diazotrophs, pot-AOA, and pot-AOB represent functional communities in field experiment 1; 2017- and 2018-diazotrophs, 2017- and 2018-AOA, and 2017- and 2018-AOB represent functional communities in field experiment 2. CK, noncolonized peanuts; P, *Ph. Liquidambaris*-colonized peanuts; PR, presowing stage; SS, seedling stage; FS, flowering stage; PS, podding stage; MS, maturing stage; black arrows, specific T-RF genotypes; red arrows, soil properties.

Redundancy analysis (RDA) was used to further elucidate the differences in functional microbial communities and their correlations with soil properties. The results of Figure 2 demonstrate that rhizosphere N transformation-related microbial communities can be significantly affected by fungal endophyte *Ph. liquidambaris* colonization, and the functional communities in PL+ were clustered far away from those in the PL- treatment group, indicating that heterogeneity could be sustained until peanut maturity. Of course, the most important finding was still the change in the seedling stage community. For the diazotroph community structure, the results presented in Figure 2a–c show that increases in the abundance and diversity of T-RF genotypes such as 51 bp, 61 bp, 153 bp, 241 bp, and 268 bp, which were identified as *Bradyrhizobium arachidis* Wang et al., 2015, *Bradyrhizobium yuanmingense* Yao et al., 2002, *Bradyrhizobium japonicum* C, and *Bradyrhizobium* sp., should be considered the most important factor affecting the overall change in the diazotroph community at the seedling stage (Tables S1–S6). However, for AOA and AOB communities, the significant inhibition effects on uncultured *crenarchaeote*, *thaumarchaeote*, archaeon (T-RF genotypes 64 bp, 71 bp, 86 bp, 122 bp, 188 bp, 421 bp, 435 bp, and 555 bp), and bacteria (65 bp, 121 bp, 169 bp, 208 bp, 270 bp, 291 bp, and 488 bp) should be considered the major reason that PL+ separately occupied independent regions within the RDA ordination plots at the seedling stage (Figure 2d–i; Tables S1–S6). Then, an even more interesting experimental result is that with the development of peanuts from the flowering to maturing stages, the population structures of diazotroph, AOA, and AOB communities were further regulated and mainly accompanied by increases in their abundances and diversities, which should serve as the primary factors causing the structural differences in N transformation-related microbial communities in PL+ when compared to the PL- treatments (Figure 2; Tables S1–S6). In addition, the dynamic changes in rhizosphere soil properties, such as TOC, DOC, DON, NH_4_^+^-N, NO_3_^−^-N, AP, AK, and PNR, showed positive correlations with the community adjustments of rhizosphere diazotrophs, AOA, and AOB in the long-term monocropped peanuts colonized by the fungal endophyte *Ph. liquidambaris* (Figure 2; Tables S7 and S8). This was especially the case for NH_4_^+^-N and NO_3_^−^-N, which may play important roles in regulating and interacting with rhizosphere N-transforming microbial communities.

### 3.4. Dynamics of Rhizosphere Soil N Availability Caused by Endophytic Fungus Phomopsis liquidambaris Colonization Involved in Promoting Peanut Nodulation and N Utilization During Different Growth Periods

**Figure 3:**
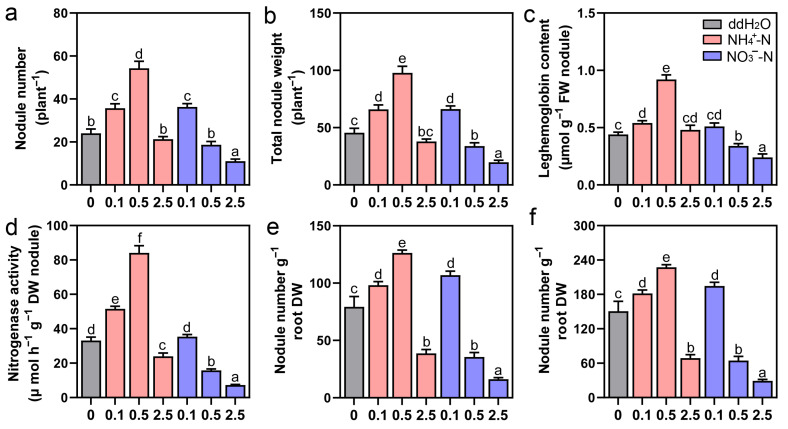
Dynamic changes in available N in rhizosphere caused by *Phomopsis liquidambaris* colonization are beneficial for peanut nodulation. The nodule-related biological parameters of peanuts were analyzed during the flowering stage under the different NH_4_^+^-N and NO_3_^−^-N concentration gradients. (**a**,**b**) Count nodule number and dry weight; (**c**,**d**) nodule N_2_ fixation capacity detection; (**e**,**f**) nodule-specific parameter analysis. Data and errors are mean ± SD (n = 3), and different letters indicate significant differences among treatments (one-way ANOVA with Tukey’s test, *p* < 0.05). Each biological replicate represents a pooled sample from at least six individual plants. NH_4_^+^-N and NO_3_^−^-N concentration units, mM.

To further confirm whether the N availability of the rhizosphere mediates peanut nodulation, we performed a controlled NH_4_^+^-N and NO_3_^−^-N addition experiment and found that when the NH_4_^+^-N concentration was lower than 0.5 mM, a higher NH_4_^+^-N concentration resulted in a higher nodule number, biomass, specific nodule parameters, and N_2_ fixation activity (Figure 3a–f). By tracking symbiotic signaling molecules, we found that 0.5 mM NH_4_^+^-N increases the production of root phytohormones, Ca^2+^, H_2_O_2_, and NO (Figure S4e–j). Similar positive results can be found in the expressions of peanut–*Bradyrhizobium* symbiosis-related genes (Figure S4a–d). In contrast, a higher NO_3_^−^-N concentration resulted a lower nodule number, biomass, specific nodule parameters, and N_2_ fixation activity. This was because 0.5 mM NO_3_^−^-N could limit the release of symbiotic signaling molecules and the expressions of symbiosis-related genes (Figure S4). These results were consistent with the findings from field experiments 1 and 2, where appropriate concentrations of rhizosphere NH_4_^+^-N and NO_3_^−^-N caused by *Ph. liquidambaris* stimulated the production of nodulation-related symbiotic signaling molecules and the expression of symbiosis-related genes, whereas higher NO_3_^−^-N may limit signal production and symbiosis gene expression (Figures S1e–h and S5a–j). In addition, as mentioned above, along with peanut development to the podding and maturing stages, the significantly enhanced NH_4_^+^-N and NO_3_^−^-N concentrations in the PL+ rhizosphere directly led to increases in N accumulation in different tissues of peanuts (Figures S1i–k, S2g–i and S3a,b,d,e). These results showed that nodulation and N utilization can be directly improved by the dynamic adjustment of rhizosphere N availability at different growth stages of peanuts colonized by the endophytic fungus *Ph. liquidambaris*.

### 3.5. Specific Root Exudate Components Triggered by Fungal Endophyte Phomopsis liquidambaris Colonization Promote Native Bradyrhizobium Nodulation

**Figure 4:**
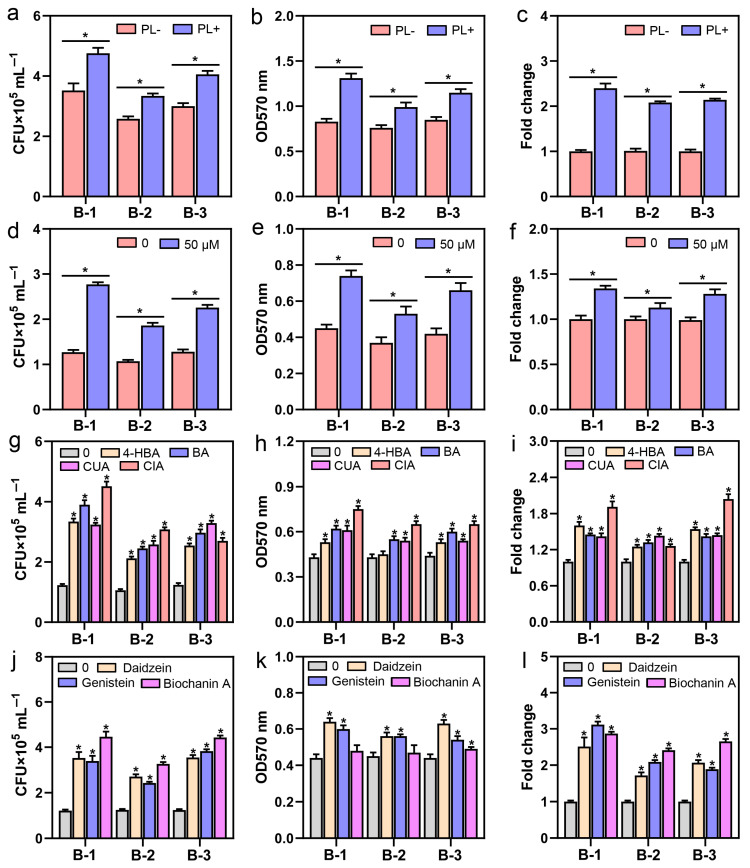
Specific root exudates from peanuts colonized by *Phomopsis liquidambaris* can enhance nodulation-related biological activities of native *Bradyrhizobium*. Effects of specific peanut root exudates (**a**–**c**), oxalic acid (**d**–**f**), phenolics (**g**–**i**), and flavonoids (**j**–**l**) derived from *Ph. liquidambaris* colonization on the chemotaxis, biofilm formation, and *nodC* gene expression of native *Bradyrhizobium* isolated from peanut nodules. Values are represented by the mean ± SD (n = 3). Each biological replicate represents the mean value of five separate experimental data. Asterisks in (**a**–**c**) indicate the significant differences between the PL- and PL+ treatments for the same *Bradyrhizobium* strain (Student’s *t*-test; *p* < 0.05). Asterisks in (**d**–**l**) indicate the significant difference between the control and the treatment of one specific root exudate component for the same *Bradyrhizobium* strain (Student’s *t*-test; *p* < 0.05). PL-, noncolonized root exudate; PL+, *Ph. Liquidambaris*-colonized root exudate; 0, ddH_2_O; 4-HBA, 4-hydroxybenzoic acid; BA, benzoic acid; CUA, coumaric acid; CIA, cinnamic acid. B-1, *Bradyrhizobium japonicum*; B-2, *Bradyrhizobium* sp.; B-3, *Bradyrhizobium liaoningense*.

To further explore the mechanism of how fungal endophyte *Ph. liquidambaris* colonization promotes peanut–*Bradyrhizobium* symbiosis at the root surface, three native *Bradyrhizobium* strands isolated from peanut nodules were used for symbiotic detection (Figure S6). The results demonstrated that root exudates collected at the seedling stage of PL+ peanuts could effectively increase the chemotaxis, biofilm formation, and *nodC* gene expression of native *Bradyrhizobium* (Figure 4a–c). This might be mainly due to the production of specific components in root exudates stimulated by *Ph. liquidambaris* colonization, including organic acid (oxalic acid), phenolics (4-hydroxybenzoic, benzoic, coumaric, and cinnamic acids) and flavonoids (daidzein, genistein, and biochanin A), because their individual application also significantly enhanced the nodulation-related physiological processes of native *Bradyrhizobium* (Figure 4d–l). By tracking the dynamic changes in exudations under the field conditions, a significant increase in phenolics and flavonoids caused by *Ph. liquidambaris* colonization was observed, which only occurred at the seedling and flowering stages. This should enhance the peanut–*Bradyrhizobium* interaction and stimulate nodulation. Meanwhile, most resources, including TC, TN, soluble sugar, amino acids, and organic acids, could maintain higher levels in PL+ than PL- throughout the entire growth stage of peanuts colonized by *Ph. liquidambaris* (Table S9). The colonization status of the fungal endophyte *Ph. liquidambaris* in roots is shown in Figure S7.

### 3.6. Endophytic Fungus Phomopsis liquidambaris Colonization Increases Overall N Utilization and Reduces N Fertilizer Inputs in Monocropped Peanuts

Compared with normal N fertilizer treatment, low-N-fertilizer application could cause higher transcriptions of symbiosis-related genes (Figure S8a–d), but the highest expressions were observed in P+BMI when compared to the BMI and P treatments, respectively. Peanut phytohormones that participate in mediating legume–rhizobia symbiosis showed low levels in the low-N-supply treatments (Figure S8e–g); however, these could be restored through BMI and P treatments, and the phytohormone levels in the co-inoculated group (P+BMI) even exceeded those in the normal N application treatment. Similarly, the signals of Ca^2+^, H_2_O_2_, and NO showed the highest concentration for P+BMI in low-N-supply soil (Figure S8h–j). Along with the dynamics of symbiosis-related gene expression and signal production, the available N status in the peanut rhizosphere varied among treatments and peanut development stages. At the nodule initiation stage, NH_4_^+^-N showed higher levels, whereas NO_3_^−^-N showed lower levels in the PL+ planting treatment groups under both low- and normal-N conditions (Figure S9d,e). This was consistent with the results showing that PL+ increased the abundance of diazotrophs but reduced rhizosphere AOA and AOB (Figure S9a–c). Furthermore, the dynamic trend of soil PNR was also consistent with the abundance changes in rhizosphere ammonia-oxidizing microorganisms (Figure S9f). However, in comparison with the non-inoculated groups, only pre-inoculation with *Bradyrhizobium* did not cause differential changes in N-transforming microbial abundance (except for the *nifH* gene) and the available N level. To summarize, the low-N-fertilizer experiment demonstrated that at the initial nodule stage of peanuts colonized by the fungal endophyte *Ph. liquidambaris*, plants’ internal physiological responses (symbiotic gene expressions and signal transduction) and rhizosphere microenvironments (functional microbial communities and N availability) both develop in a favorable direction for peanut–*Bradyrhizobium* symbiosis interaction.

**Figure 5:**
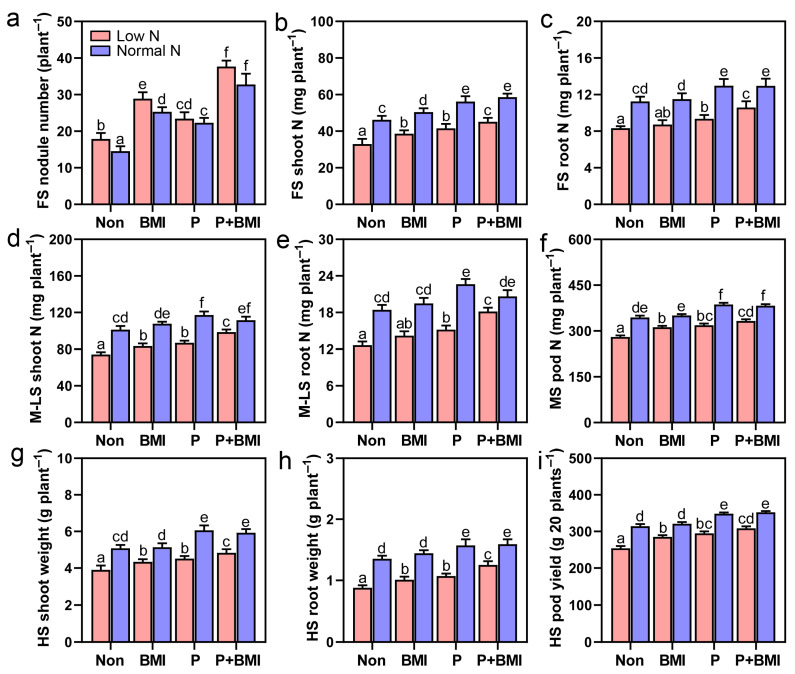
Co-inoculation of *Phomopsis liquidambaris* with native *Bradyrhizobium* increases nodulation, N_2_ fixation, and the biomass of continuous cropping peanuts under low-N-fertilizer conditions (field experiment 3). (**a**) The number of peanut nodules at the flowering stage; (**b**–**e**) N accumulation in the shoots and roots of peanuts at the flowering and mid–late stages; (**f**) pod N accumulation of peanuts at maturing stage; and (**g**–**i**) peanut agronomic characteristics at harvest stage. Data and errors are mean ± SD (n = 3), and different letters indicate significant differences among treatments (one-way ANOVA with Tukey’s test, *p* < 0.05). Each biological replicate represents a pooled sample from at least six individual plants. Non, non-inoculated seedlings; BMI, seedlings only inoculated with BMI (*Bradyrhizobium* mixed inoculant); P, seedlings only inoculated with *Ph. liquidambaris*; P+BMI, seedlings co-inoculated with *Ph. liquidambaris* and BMI; FS, flowering stage; M-LS, mid–late stage; HS, harvest stage; Low or Normal N, low or normal N fertilizer.

**Figure 6:**
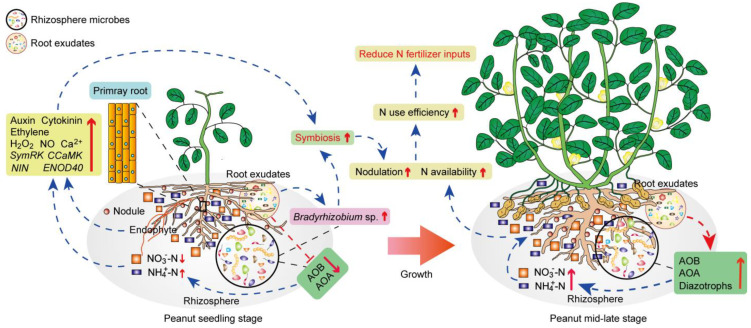
A model of the endophytic fungus *Phomopsis liquidambaris* stimulating nodulation and overall N utilization of long-term monocropped legume peanuts throughout the entire growth stage. At the seedling stage, the colonization of *Ph. liquidambaris* can effectively inhibit the abundances of peanut rhizosphere AOA and AOB, which causes transient rhizosphere NH_4_^+^-N accumulation, accompanied by a reduction in NO_3_^−^-N. This fine adjustment of rhizosphere N availability (NH_4_^+^-N and NO_3_^−^-N concentrations) can significantly enhance the nodulation-related phytohormones levels (auxin, cytokinin, and ethylene), symbiotic signaling molecule transmissions (Ca^2+^, H_2_O_2_, and NO), and nodulation-related gene expressions (*SymRK*, *CCaMK*, *NIN*, and *ENOD40*) in host peanuts. Simultaneously, the peanut rhizosphere native *Bradyrhizobium* spp. was enhanced, and their nodulation-related biological activities were stimulated by the derived root exudates (organic acid, phenolic, and flavonoid compounds) caused by *Ph. liquidambaris* colonization. All of these contribute to enhancing peanut–*Bradyrhizobium* interactions and initiating the formation of nodules at the seedling stage, and then they also increase host N_2_ fixation. With the increase in peanut growth stages, *Ph. liquidambaris* colonization again reshaped the community of rhizosphere N-transforming microbiota (diazotrophs, AOA and AOB) and resulted in an increase in soil N availability (NH_4_^+^-N and NO_3_^−^-N) at the crucial mid–late growth stage. This was mainly because with the growth of peanuts, the concentrations of phenolics and flavonoids in root exudates decreased, which reduced the inhibitory effects on rhizosphere ammonia-oxidizing microorganisms. Meanwhile, the increased secretions of TC, TN, sugars, and amino acids may serve as rich C and N resources for the reproduction of rhizosphere diazotrophs, AOA, and AOB. Therefore, our proposed model shows that the introduction of the fungal endophyte *Ph. liquidambaris* may be an effective measure for maintaining the yield of long-term monocropped legume peanuts by promoting nodulation at an early stage and improving N availability at the mid–late stage. This may help legume crops to reduce the input of N fertilizer and facilitate the development of sustainable agriculture. Red upwards arrows, significant upregulation; red downwards arrows, significant downregulation; dotted blue or red lines with arrows, positive effects; dotted red line ending with a short bar, negative effects; dotted black lines, subordination relationship.

Further nodulation parameter analysis showed that the PL+ peanuts pre-inoculated with *Bradyrhizobium* indeed presented the highest nodule number, nodule dry weight, specific nodule parameters, and N_2_ fixation capacity in low-N conditions (Figure 5a, Table S10), which should be considered the most important factor for enhancing N acquisition at the flowering stage when compared to the other treatments (Figure 5b,c). When peanuts grow to the important mid–late stage, the results in Figure S9g–i demonstrate that under low-N-fertilizer conditions, the abundances of rhizosphere diazotrophs, AOA, and AOB in the treatment of PL+ (or PL+BMI) are significantly higher than those in the PL- rhizosphere. This caused significant increases in rhizosphere NH_4_^+^-N, NO_3_^−^-N, and PNR levels (Figure S9j–l), which was helpful for the absorption of N by peanuts and obviously resulted in the highest shoot, root, and pod N accumulation in PL+ peanuts during the crucial podding period (Figure 5d–f). Finally, the most important result was that obtained when *Ph. Liquidambaris*-colonized seedlings were reinoculated with native *Bradyrhizobium*. The biomass and yield of peanuts under low-N conditions increased significantly and similarly to the normal N supply treatment (Figure 5g–i). Therefore, these results indicate that *Ph. liquidambaris* should have practical application potential for alleviating the reduction in the continuous cropping of peanuts by increasing nodulation and overall N utilization. This helps to reduce N fertilizer inputs, and an underlying mechanism for the fungal endophyte *Ph. liquidambaris* promoting the nodulation and overall N use of legume peanuts is proposed in Figure 6.

## 4. Discussion

In an intensive agroecosystem, maximizing N_2_ fixation and exogenous N utilization is critical to address the three challenges of food security, environmental degradation, and climate change [1]. In long-term monocropped grain legumes, a delicate balance exists between applying N fertilizer to boost yield and maintaining an optimal number of nodules. The excessive use of N fertilizer disrupts this equilibrium, reduces N use efficiency, and contributes to serious environmental pollution [1,8,35]. Therefore, enhancing nodulation and N utilization under low-N-fertilizer input conditions is critical for sustainable legume production [8,35]. Endophytes represent a promising microbial resource with demonstrated benefits in agricultural systems, including the potential to enhance N_2_ fixation in host plants [36,37]. However, no studies have yet reported how introducing endophytes can jointly regulate both nodulation and N utilization throughout the entire growth cycle of long-term monocropped grain legumes under limited N fertilization. Our previous studies have shown that the endophytic fungus *Ph. liquidambaris* significantly improves nodulation, N accumulation, and rhizosphere N availability in peanut systems [20,21], indicating its potential to balance nodulation and N utilization. However, the strategic mechanisms through which *Ph. liquidambaris* orchestrates these effects remain to be fully elucidated.

Enhancing nodulation and N_2_ fixation represents a crucial strategy for maintaining nitrogen acquisition in long-term monocropped grain legumes under low N fertilization [8]. In the present study, two field experiments (1 and 2) consistently demonstrated that colonization by the fungal endophyte *Ph. liquidambaris* significantly increased nodulation traits and N_2_ fixation capacity in continuously cropped peanut, corroborating previous reports of its beneficial role in peanut–*Bradyrhizobium* symbiosis [20,22]. Nodule formation in legumes relies on optimal physiological conditions and complex molecular dialogue between the host plants and rhizobia [3,9]. While substantial progress has been made in understanding nodulation mechanisms in model legumes such as *Medicago truncatula* Gaertn. and *Lotus japonicus* (Regel) K.Larsen [11], the genetic complexity of cultivated peanut has hindered the development of molecular tools and limited insight into its symbiotic interaction with *Bradyrhizobium* [19]. Although several studies have reported that co-inoculation with endophytes can improve legumes’ symbiotic performance, most have focused on phenomenological observations under controlled greenhouse conditions, with limited mechanistic exploration in realistic field environments [37,38]. In this context, elucidating the microecological mechanisms by which fungal endophytes promote nodulation in monocropped legumes represents an important research direction.

In this study, we first discovered that colonization by the fungal endophyte *Ph. liquidambaris* significantly suppressed the abundance and diversity of ammonia-oxidizing archaea (AOA) and bacteria (AOB) in the rhizosphere of long-term monocropped peanuts at the seedling stage. This suppression led to a transient accumulation of ammonium (NH_4_^+^-N) and a concomitant reduction in nitrate (NO_3_^−^-N) concentration. Such fine-tuning of rhizosphere N availability effectively enhanced nodulation-related phytohormone levels, symbiotic signaling transduction, and the expression of symbiosis-related genes in host peanuts. Previous studies have demonstrated that high NO_3_^−^-N concentrations strongly inhibit the nodule initiation of legumes, whereas NH_4_^+^-N—within a certain threshold—can promote the transcription expressions of genes that are essential for nodule initiation [15,16,39]. Concurrently, we observed an enrichment of *Bradyrhizobium* sp. in the rhizosphere, whose nodulation-related activities were significantly stimulated by specific phenolic and flavonoid compounds in root exudates induced by *Ph. liquidambaris* colonization. This enhancement in root–rhizobia communication improved infection efficiency and facilitated the establishment of symbiosis [10,40]. Root exudates are known to shape microbial community structure and function in the rhizosphere [41], with certain secondary metabolites influencing nitrogen cycling processes [41,42]. Notably, phenolics and flavonoids have been shown to inhibit ammonia-oxidizing microorganisms [42,43], leading to reduced nitrate NO_3_^−^-N levels. Our observation of increased phenolic and flavonoid secretion in *Ph. liquidambaris*-colonized seedlings supports this mechanism, highlighting a dual role of root exudates in simultaneously enhancing *Bradyrhizobium* activity and suppressing ammonia oxidizers. Therefore, all of these progressively increasing effects can be used to explain why fungal endophyte *Ph. liquidambaris* colonization can increase *Bradyrhizobium* symbiosis and nodulation of long-term monocropped peanuts.

While high yield represents a primary objective in grain legume cultivation, the nitrogen supplied through nodulation and N_2_ fixation often proves insufficient to meet crop demands throughout the entire growth cycle [8]. Therefore, enhancing N utilization—particularly during the mid–late growth stages—is critical for maintaining yield and quality in long-term monocropped legumes [1]. Although researchers hope to increase N use efficiency by improving traditional agronomic practices or gaining an enhanced understanding of modern bioengineering technologies, these methods still face many huge and unknown challenges, and they are difficult to adopt widely because of limitations imposed by techniques, time and cost factors, and unstable effects [35,44,45]. Plant-beneficial rhizosphere microorganisms are those that are able to colonize the rhizosphere and improve N utilization by means of a wide variety of mechanisms, like organic matter mineralization, biological N_2_ fixation, improved soil N availability, biocontrol against soil-borne pathogens, etc. [12]. Specifically, the abundance and diversity of N-transforming microorganisms—such as diazotrophs, ammonia-oxidizing archaea (AOA), and ammonia-bacteria (AOB)—directly influence the rhizosphere’s biological N_2_ fixation and nitrification process, thereby reflecting soil N availability [14,42,43]. In addition to the deterioration of a variety of beneficial microbial communities in the rhizosphere, the long-term monoculture of peanuts also resulted in a significant imbalance in the microflora of rhizosphere diazotrophs, AOA, and AOB, causing a marked decrease in the soil N metabolism and available N level [6,46]. Rebuilding these rhizosphere microbial communities, particularly those involved in N transformation, may thus provide a viable strategy for increasing N utilization throughout the peanut growth cycle. Interestingly, as the peanuts grew from the flowering to maturing stages, we found an opposite phenomenon to that observed in the seedling period; that is, the colonization of *Ph. liquidambaris* significantly enhanced the abundances and diversities of rhizosphere diazotrophs, AOA, and AOB, accompanied by enhanced rhizosphere soil NH_4_^+^-N and NO_3_^−^-N concentrations. This reversal may be attributed to changes in root exudation patterns: as peanuts grew to the mid–late stage, phenolic and flavonoid compounds decreased, while total carbon (TC), nitrogen (TN), sugars, and amino acids increased, providing favorable resources for the proliferation of nitrogen-cycling microorganisms [28,41,47]. Therefore, our study provides the first evidence that an exogenous beneficial endophyte not only promotes legume crop early-stage nodulation but also significantly increases the level of rhizosphere available N at the mid–late growth stage, which may help to directly increase the N utilization of long-term monocropped grain legumes throughout the entire growth period.

The production of N fertilizer consumes 1–2% of global energy, but crops use only 30–50% of the applied N [45]. The excessive use of N fertilizer increases the cost of crop production, as well as causing severe environmental problems [1]. Enhancing N use efficiency is therefore critical for sustainable agriculture [35]. Tripartite or multiple interactions between legumes and their symbionts are important for host nodulation and growth [23]. While rhizobial inoculation or co-inoculation with plant growth-promoting microorganisms has been shown to improve nodulation and N_2_ fixation [5,37,38], there are still doubts about this beneficial effect, its effectiveness, the stability of the inoculants over time and under varying climatic conditions, especially since it is difficult to actually reduce the application of N fertilizer and maintain stable crop yields [48]. As a symbiotic fungus, *Ph. liquidambaris* has been reported to colonize the roots of peanuts [21]. In this study, we found that fungal colonization could significantly increase the number of nodules and specific nodulation parameters under the low-N conditions of the N fertilizer addition experiment. This was largely attributed to the peanut plants’ improved physiological responses and the rhizosphere’s microecological environment. The strongest promotion of nodulation occurred when peanuts were pre-inoculated with native *Bradyrhizobium* strains, consistent with reports that locally adapted rhizobia often exhibit superior symbiotic performance [24]. Surprisingly, an obviously synergistic effect between *Ph. liquidambaris* and *Bradyrhizobium* was observed, because co-inoculation further stimulated the nodulation potential of peanuts. This might be because the reinoculation provides sufficient rhizosphere *Bradyrhizobium* populations and depends on adjustments of the nodulation-related physiological states of hosts and *Bradyrhizobium* caused by *Ph. liquidambaris* colonization. However, at present, it is still not clear how fungal endophyte *Ph. liquidambaris* colonization and co-inoculation with *Bradyrhizobium* can be used to balance peanut nodulation and nodule autoregulation. Consistent with the mechanism mentioned in the two field experiments (1 and 2), when peanuts grow to the crucial mid–late stage, the colonization of the fungal endophyte *Ph. liquidambaris* can also further enhance rhizosphere soil N availability by improving N transformation-related microbial communities so as to ensure the N acquisition of peanuts at the maturating stage under low-N-fertilizer-supply conditions.

Our study clearly demonstrates that the co-inoculation of *Ph. liquidambaris* and *Bradyrhizobium* produced synergistic benefits for peanut growth. Notably, the peanut agronomic indices and yield under these co-inoculated low-N-fertilizer conditions were the same as in the group with only normal N fertilizer application. This result confirms the possibility of maintaining yield stability while improving N utilization in long-term monocropped legumes under low-N conditions, and potential mechanisms caused by the endophytic fungus *Ph. liquidambaris* are proposed in Figure 6. Compared to our previous results, this work provides the first evidence elucidating the biological mechanisms behind the fungal endophyte *Ph. liquidambaris* promoting N utilization in long-term monocropped peanuts from the new perspectives of plant physiology and soil microecology. While previous studies have reported that *Ph. liquidambaris* can improve photosynthesis and stress resistance, the rhizosphere bacteria–fungi ratio, bacterial metabolic function, soil enzyme activities, and nutrient status and degrade toxic allelochemicals [5,21], alongside potentially facilitating flavonoid secretion or the transportation of *Bradyrhizobium* to the rhizosphere [23], the current study establishes a more comprehensive mechanism. In this study, our results further confirmed that the colonization of the fungal endophyte *Ph. liquidambaris* can improve the nodulation and N availability of legume crops in long-term monocropped systems throughout the entire growth stage, and we proposed unprecedented underlying mechanisms, which might be beneficial for increasing crops’ N acquisition and reducing N fertilizer inputs under field conditions.

## 5. Conclusions

Our study revealed that the colonization of the fungal endophyte *Ph. liquidambaris* has positive impacts on peanut root nodulation and rhizosphere N availability throughout the entire growth stage. A possible mechanism behind this from plant physiological and soil ecological perspectives is that *Ph. liquidambaris* inhibits the growth of rhizosphere AOA and AOB. This biological limitation modulates the balance of rhizosphere NO_3_^−^-N and NH_4_^+^-N, enhancing the nodulation-related physiological responses of host peanuts. Simultaneously, due to the enrichment of rhizosphere *Bradyrhizobium* sp., high nodulation-related biological activities were stimulated by the root exudates triggered by fungal colonization. All of these factors contributed to the enhancement of peanut–*Bradyrhizobium* symbiosis interaction and the initiation of nodulation. Along with peanut growth, *Ph. liquidambaris* colonization further reshaped the community of rhizosphere diazotrophs, AOA, and AOB, which enhanced soil N availability for peanut utilization during the mid–late period. Finally, the low-N-fertilizer application experiment further confirmed that the colonization of the fungal endophyte *Ph. liquidambaris* can be used as an effective method to maintain the yield of long-term monocropped legume crops by promoting nodulation at an early stage and improving rhizosphere N availability in the mid–late stage. Our results may help to reduce N fertilizer inputs and facilitate the development of sustainable agriculture.

## Data Availability

The original contributions presented in this study are included in the article/Supplementary Material. Further inquiries can be directed to the corresponding authors.

## Supplementary Materials

The following supporting information can be downloaded at https://www.mdpi.com/article/10.3390/jof12010065/s1: Figure S1: *Phomopsis liquidambaris* colonization significantly increases the nodulation, N_2_ fixation, and plant biomass of continuous cropping peanuts in field experiment 1; Figure S2: *Phomopsis liquidambaris* colonization significantly increases the nodulation, N_2_ fixation, and plant biomass of continuous cropping peanuts in field experiment 2. Figure S3: *Phomopsis liquidambaris* colonization significantly affects the level of available N in the rhizosphere. Dynamic changes in NH_4_^+^-N and NO_3_^−^-N concentrations and soil PNR in the peanut rhizosphere for field experiment 1 and field experiment 2 throughout the entire growth stage. Figure S4: Dynamic changes in available N in the rhizosphere caused by *Phomopsis liquidambaris* colonization are beneficial for peanut–*Bradyrhizobium* symbiosis. Figure S5: *Phomopsis liquidambaris* colonization significantly enhances peanut–*Bradyrhizobium* symbiosis in field experiment 2. Figure S6: Effects of inoculation of native *Bradyrhizobium* on nodule parameters and shoot and root N contents of peanuts at the flowering stage. Figure S7: Colonization of the fungal endophyte *Phomopsis liquidambaris* in the roots of peanuts throughout the entire growth stage in field experiment 1. Figure S8: Co-inoculation of *Phomopsis liquidambaris* with native *Bradyrhizobium* enhances peanut–*Bradyrhizobium* symbiosis in continuous cropping peanuts under low-N-fertilizer conditions. The symbiosis-related gene expressions of *SymRK*, *CCaMK*, *NIN*, and *ENOD40* and the levels of nodulation signaling molecules were analyzed in the roots at the nodule initiation stage. Figure S9: Co-inoculation of *Ph. liquidambaris* with native *Bradyrhizobium* improves rhizosphere N-transforming microbial abundance and available N level throughout the growth of peanuts under low-N-fertilizer conditions (field experiment 3). Table S1: Field experiment 1 design; relative abundances of T-RFs of *nifH* (diazotrophs), *arch-amoA* (AOA), and *amoA* (AOB) genes restricted by *Hae*III and *Mbo*I, respectively, in the rhizosphere soils. Table S2: The partial sequence identities of T-RFs obtained from rhizosphere soil diazotroph, AOA, and AOB community clone libraries (field experiment 1 design). Table S3: Shannon index (*H*) and evenness index (*E*) of diazotroph, AOA, and AOB communities calculated from T-RFLP data (field experiment 1 design). Table S4: Field experiment 2 design; relative abundances of T-RFs of *nifH* (diazotrophs), *arch-amoA* (AOA), and *amoA* (AOB) genes restricted by *Hae*III and *Mbo*I, respectively, in the rhizosphere soils. Table S5: The partial sequence identities of T-RFs obtained from rhizosphere soil diazotroph, AOA, and AOB community clone libraries (field experiment 2 design). Table S6: Shannon index (*H*) and evenness index (*E*) of diazotroph, AOA, and AOB communities calculated from T-RFLP data (field experiment 2 design). Table S7: Soil properties of rhizosphere throughout the entire growth period of peanuts (field experiment 1 design). Table S8: Soil properties in rhizosphere throughout the entire growth period of peanuts (field experiment 2 design). Table S9: Determination of the dynamic changes in root exudate components throughout the entire growth period of peanuts (field experiment 1 design). Table S10: Effects of *Phomopsis liquidambaris* colonization and co-inoculation with native *Bradyrhizobium* on peanut nodulation parameters under the different N fertilizer conditions (field experiment 3 design).
