# Supplementary material for: Fungal Endophyte Comprehensively Orchestrates Nodulation and Nitrogen Utilization of Legume Crop (Arachis hypogaea L.)
Source: J Fungi (Basel). 2026 Jan 13;12(1):65. doi: 10.3390/jof12010065 (PMC12843411; doi:10.3390/jof12010065)
Supplement: Supplementary file 1 [file jof-12-00065-s001.zip › jof-4060727-supplementary.pdf]

Supporting Information

Fungal endophyte comprehensively orchestrates nodulation and nitrogen utilization of legume crop (*Arachis hypogaea* L.)

Xing-Guang Xie[1,2], Kai Sun[1], Hui-Jun Jiang[1], Yuan-Yuan Zhao[1], Xiao-Gang Li[4], Ting Han[2,*], Yan Chen[3,*], Chuan-Chao Dai[1,*]

[1]Jiangsu Key Laboratory for Microbes and Functional Genomics, Jiangsu Engineering and Technology Research Center for Industrialization of Microbial Resources, College of Life Sciences, Nanjing Normal University, Nanjing, Jiangsu Province, 210023, People's Republic of China.
[2]Deparment of Pharmacognosy, School of Pharmacy, Naval Medical University, Shanghai 200433, People's Republic of China.
[3]State Key Laboratory of Soil and Sustainable Agriculture, Institute of Soil Science, Chinese Academy of Sciences, Nanjing, Jiangsu Province 210008, People's Republic of China.
[4]Co-Innovation Center for Sustainable Forestry in Southern China, College of Biology and the Environment, Nanjing Forestry University, Nanjing 210037, People's Republic of China.

[*]Corresponding author: Chuan-Chao Dai
E-mail address: daichuanchao@njnu.edu.cn
Tel./fax: +86 25 85891382
Corresponding author at: No. 1, Wenyuan Road, Nanjing Normal University, Qixia District, Nanjing, Jiangsu Province, 210023, China.

[*]Co-corresponding author: Yan Chen
E-mail address: chenyan@issas.ac.cn

[*]Co-corresponding author: Ting Han
E-mail address: hanting@smmu.edu.cn

Table S1 Field experiment 1 design, relative abundances of T-RFs of *nifH* (diazotrophs), *arch-amoA* (AOA) and *amoA* (AOB) genes restricted by *Hae*III and *Mbo*I, respectively, in the rhizosphere soils

**Items**	**T-RFs (bp)**	**PR-PL-**	**PR-PL+**	**SS-PL-**	**SS-PL+**	**FS-PL-**	**FS-PL+**	**PS-PL-**	**PS-PL+**	**MS-PL-**	**MS-PL+**
	**51**	/	/	/	3.05 (0.09)b	1.35 (0.03)a	2.18 (0.08)b	1.15 (0.06)a	1.50 (0.08)b	/	/
	**61**	/	/	/	2.39 (0.08)b	/	1.63 (0.07)		1.35 (0.08)	/	1.60 (0.03)
	**96**	14.70 (0.92)a	14.02 (1.53)a	8.62 (0.25)b	5.24 (0.08)a	12.07 (0.62)b	8.79 (0.26)a	10.70 (0.85)a	15.17 (0.77)b	9.92 (0.29)a	13.12 (0.83)b
	**153**	/	/	/	1.47 (0.11)	/	1.18 (0.09)	/	1.09 (0.04)	/	1.15 (0.03)
nifH	**163**	8.15 (0.33)a	8.71 (0.18)a	5.39 (0.07)b	3.80 (0.07)a	10.56 (0.32)b	6.76 (0.26)a	12.19 (1.56)b	7.54 (0.47)a	8.51 (0.41)b	7.21 (0.15)a
	**241**	/	/	12.65 (0.09)a	18.36 (0.91)b	9.15 (0.27)a	13.90 (0.30)b	8.25 (0.08)a	11.04 (0.48)b	12.44 (1.13)a	15.28 (0.55)b
	**268**	11.90 (1.09)a	11.67 (1.01)a	18.11 (1.15)a	28.41 (0.69)b	14.75 (1.09)a	20.62 (1.97)b	13.50 (0.52)a	18.00 (1.49)b	14.32 (0.62)a	19.68 (0.74)b
	**338**	/	/	/	1.80 (0.06)	/	1.08 (0.03)	/	2.38 (0.08)	/	1.66 (0.07)
	**438**	54.19 (2.03)a	55.99 (1.07)a	44.19 (0.98)b	26.55 (2.00)a	42.55 (0.78)b	36.05 (1.04)a	44.08 (1.13)b	33.10 (1.47)a	44.06 (2.96)b	31.24 (1.23)a
	**58**	1.61 (0.06)a	1.63 (0.04)a	3.10 (0.30)	/	/	/	/	/	/	/
	**64**	/	/	1.24 (0.07)	/	/	/	/	/	/	/
	**73**	1.43 (0.06)a	1.44 (0.06)a	1.57 (0.08)a	4.31 (0.18)b	1.61 (0.05)a	5.11 (0.41)b	4.25 (0.08)b	3.45 (0.21)a	2.48 (0.36)a	2.47 (0.22)a
	**86**	/	/	1.12 (0.03)	/	/	/	/	/	/	/
	**122**	/	/	1.25 (0.24)	/	/	/	/	/	/	/
	**137**	1.01 (0.01)a	1.08 (0.04)a	3.02 (0.09)a	3.64 (0.22)b	2.01 (0.13)a	3.12 (0.09)b	1.84 (0.07)a	7.72 (0.32)b	2.56 (0.20)a	6.91 (0.69)b
arch-amoA	**188**	/	/	1.71 (0.17)	/	/	/	/	/	/	/
	**306**	/	/	/	/	/	1.41 (0.11)	/	1.19 (0.11)	/	/
	**329**	61.68 (2.10)a	64.19 (1.53)a	45.50 (0.91)a	44.20 (1.86)a	31.46 (1.06)a	35.48 (2.67)b	29.82 (2.50)a	34.87 (0.17)b	33.00 (2.88)a	40.77 (1.81)b
	**421**	16.91 (0.27)a	17.16 (1.29)a	22.35 (1.63)a	34.60 (3.49)b	53.93 (2.59)b	41.34 (4.87)a	52.21 (1.88)b	39.37 (1.68)a	50.25 (1.82)b	33.55 (2.48)a
	**431**	/	/	/	/	/	/	/	/	/	2.58 (0.16)

	**443**	1.87 (0.32)a	1.88 (0.14)a	2.83 (0.27)a	3.00 (0.24)a	3.50 (0.70)a	4.74 (0.21)b	2.17 (0.20)a	3.88 (0.06)b	3.95 (0.18)a	6.92 (0.18)b
	**491**	/	/	/	/	/	1.10 (0.05)	/	/	/	/
	**555**	/	/	1.45 (0.09)a	1.31 (0.09)a	/	/	/	1.01 (0.06)	/	1.25 (0.19)
	**109**	61.55 (2.88)a	62.71 (1.48)a	54.23 (1.68)a	55.02 (1.81)a	66.89 (1.80)b	44.89 (2.31)a	67.48 (0.70)b	41.11 (1.30)a	65.58 (1.14)b	35.74 (0.57)a
	**121**	/	/	2.23 (0.23)	/	/	6.02 (0.11)	/	4.70 (0.13)	/	3.53 (0.45)
amoA	**169**	/	/	3.42 (0.29)	/	2.53(0.17)a	4.79 (0.14)b	1.68 (0.06)a	5.98 (0.18)b	2.59 (0.05)a	5.90 (0.15)b
	**208**	3.38 (0.10)a	3.25 (0.10)a	5.49 (0.29)b	2.52 (0.09)a	4.16 (0.25)a	7.56 (0.25)b	/	8.81 (0.18)	/	10.28 (0.31)
	**280**	27.62 (1.61)a	26.07 (2.39)a	25.22 (1.25)a	35.47 (0.81)b	20.88 (1.11)a	27.31 (1.77)b	25.14 (0.79)a	31.88 (1.39)b	24.97 (1.52)a	37.70 (1.05)b
	**291**	/	/	1.42 (0.10)	/	/	/	/	/	/	/

Mean values and SDs of three biological replicates are presented, with each biological replicate representing a pooled sample from at least six individual rhizosphere soils. Values in the same row followed by different letters (a, b) differed significantly between different treatments (PL- and PL+ treatments) at the same sampling stage, and the same letters indicate no significant difference (Student's *t*-test; $p < 0.05$). PL-, noncolonized peanuts; PL+, *P. liquidambaris* colonized peanuts. PR, presowing stage; SS, seedling stage; FS, flowering stage; PS, podding stage; MS, maturing stage.

Table S2 The partial sequence identities of obtained T-RFs from rhizosphere soil diazotrophs, AOA and AOB community clone libraries (field experiment 1 design)

Items	T-RFs (bp)	GenBank accession no.	Closest match from GenBank	Coverage/ID (100%)	Number of clones
Diazotrophs	**51**	GU433551.1	*Bradyrhizobium arachidis*	91/100	12
		KM052355.1	*Bradyrhizobium* sp.	99/100	5
	**61**	FJ514070.1	*Bradyrhizobium yuanmingense*	98/99	8
	**153**	LC367311.1	*Bradyrhizobium* sp.	95/100	2
	**241**	EU544201.1	Uncultured bacterium	100/98	15
		KT027868.1	*Bradyrhizobium* sp.	100/99	4
		KY020288.1	*Bradyrhizobium japonicum*	99/100	8
	**268**	KY990684.1	*Bradyrhizobium* sp.	99/99	3
		JF834153.1	*Bradyrhizobium liaoningense*	98/99	6
		EU622091.1	*Bradyrhizobium yuanmingense*	98/100	9
	**438**	FJ466689.1	Uncultured bacterium	100/99	12
		FJ466700.1	Uncultured bacterium	100/98	35
		KC412126.1	Uncultured bacterium	100/100	10
		EU544219.1	Uncultured bacterium	100/93	21
AOA	**73**	KY118429.1	Uncultured *crenarchaeote*	100/100	5
	**137**	KU937458.1	Uncultured archaeon	99/100	4
	**188**	KC010590.1	Uncultured *thaumarchaeote*	99/92	22
	**329**	MF566524.1	Uncultured archaeon	100/99	18
		FN691275.1	Uncultured *crenarchaeote*	93/97	32
		GU396238.1	Uncultured archaeon	99/100	20
	**421**	KM116942.1	Uncultured archaeon	100/99	25
	**443**	HQ896059.1	Uncultured *crenarchaeote*	100/100	14
		AB545943.1	Uncultured *crenarchaeote*	99/99	8
AOB	**109**	KY950346.1	Uncultured bacterium	100/96	26
		KC773940.1	Uncultured bacterium	100/99	18
		MF509653.1	Uncultured bacterium	94/100	32
	**169**	GU931354.1	Uncultured bacterium	94/100	5
	**208**	KM250656.1	Uncultured bacterium	100/98	18
	**280**	GU931343.1	Uncultured bacterium	98/95	22
		KC311324.1	Uncultured bacterium	100/99	18
		MG658112.1	Uncultured bacterium	100/99	10

Table S3 Shannon index (*H*) and Evenness index (*E*) of diazotrophs, AOA and AOB communities calculated from T-RFLP data (field experiment 1 design)

**Items**		**PR**		**SS**		**FS**		**PS**		**MS**	
		H	***E***	***H***	***E***	***H***	***E***	***H***	***E***	***H***	***E***
**Diazotrophs**	**PL-**	1.69 (0.03)a	1.51 (0.03)a	1.34 (0.01)a	2.16 (0.02)a	1.46 (0.01)a	2.62 (0.02)a	1.43 (0.02)a	2.79 (0.03)a	1.38 (0.02)a	2.23 (0.03)a
	**PL+**	1.07 (002)a	1.49 (0.03)a	1.70 (0.01)b	3.72 (0.03)b	1.67 (0.01)b	3.67 (0.01)b	1.73 (0.01)b	3.59 (0.02)b	1.68 (0.01)b	3.01 (0.02)b
**AOA**	**PL-**	0.83 (0.02)a	0.47 (0.01)a	1.44 (0.03)b	0.58 (0.01)a	0.96 (0.03)a	0.60 (0.02)a	0.99 (0.02)a	0.62 (0.01)a	1.03 (0.01)a	0.64 (0.01)a
	**PL+**	0.82 (0.01)a	0.46 (0.01)a	1.16 (0.03)a	0.65 (0.01)b	1.27 (0.02)b	0.65 (0.01)b	1.30 (0.01)b	0.67 (0.01)b	1.36 (0.04)b	0.70 (0.02)b
**AOB**	**PL-**	0.75 (0.02)a	0.68 (002)a	1.11 (0.02)b	0.62 (0.01)a	0.81 (0.02)a	0.59 (0.02)a	0.66 (0.01)a	0.60 (0.01)a	0.70 (0.01)a	0.64 (0.01)a
	**PL+**	0.74 (0.02)a	0.67 (0.02)a	0.78 (0.01)a	0.71 (0.01)b	1.25 (0.01)b	0.78 (0.01)b	1.28 (0.01)b	0.80 (0.01)b	1.28 (0.02)b	0.79 (0.01)b

Mean values and SDs of three biological replicates are presented, with each biological replicate representing a pooled sample from at least six individual rhizosphere soils. Values in the same column followed by different letters (a, b) differed significantly between different treatments (PL- and PL+ treatments) at the same sampling stage, and the same letters indicate no significant difference (Student's *t*-test; $p < 0.05$). PL-, noncolonized peanuts; PL+, *P. liquidambaris* colonized peanuts. PR, presowing stage; SS, seedling stage; FS, flowering stage; PS, podding stage; MS, maturing stage.

Table S4 Field experiment 2 design, relative abundances of T-RFs of *nifH* (diazotrophs), *arch-amoA* (AOA) and *amoA* (AOB) genes restricted by *Hae*III and *Mbo*I, respectively, in the rhizosphere soils

**Items**	**T-RFs (bp)**	**PR-PL-**	**PR-PL+**	**SS-PL-**	**SS-PL+**	**FS-PL-**	**FS-PL+**	**PS-PL-**	**PS-PL+**	**MS-PL-**	**MS-PL+**
	**58**	6.94 (0.74)a	7.54 (0.51)a	12.80 (1.01)a	12.00 (0.56)a	8.56 (0.31)a	10.07 (0.54)b	6.65 (0.17)a	9.45 (0.12)b	6.34 (0.13)a	10.34 (0.63)b
	**96**	56.34 (1.63)a	55.82 (1.26)a	42.97 (2.48)b	30.58 (1.45)a	45.53 (0.99)b	29.30 (1.30)a	50.20 (1.72)b	23.73 (0.51)a	45.94 (2.16)b	35.51 (1.17)a
	**122**	1.26 (0.08)a	1.36 (0.07)a	3.63 (0.13)b	2.58 (0.28)a	4.01 (0.21)a	5.57 (0.44)b	2.31 (0.04)a	6.48 (0.16)b	3.83 (0.08)a	6.16 (0.08)b
nifH	**126**	/	/	1.28 (0.08)	/	1.09 (0.04)a	1.64 (0.10)b	1.05 (0.03)a	2.58 (0.04)b	/	/
**(2017)**	**153**	/	/	/	5.70 (0.21)	/	4.47 (0.13)	/	5.22 (0.14)	/	4.50 (0.11)
	**188**	22.50 (2.07)a	22.21 (1.01)a	15.87 (0.75)b	11.25 (0.71)a	16.46 (0.96)b	12.89 (0.30)a	18.36 (1.15)b	14.02 (0.17)a	24.03 (1.27)b	14.84 (0.60)a
	**237**	/	/	5.07 (0.14)b	3.77 (0.08)a	2.62 (0.07)a	3.50 (0.08)b	3.45 (0.12)a	8.65 (0.18)b	3.28 (0.09)a	6.20 (0.13)b
	**241**	/	/	/	9.04 (0.22)	1.21 (0.04)a	6.22 (0.26)b	2.67 (0.15)a	5.26 (0.14)b	2.26 (0.07)a	5.59 (0.13)b

	**268**	4.07 (0.09)a	4.09 (0.15)a	10.41 (0.95)a	16.05 (0.81)b	12.09 (0.48)a	14.66 (0.57)b	6.67 (0.20)a	12.10 (0.42)b	5.98 (0.17)a	8.48 (0.22)b
	**450**	/	/	/	/	/	2.44 (0.19)	/	4.29 (0.08)	/	/
	**58**	7.28 (0.41)a	7.07 (0.40)a	8.50 (0.21)a	8.39 (0.32)a	10.54 (0.57)a	12.76 (0.57)b	5.60 (0.19)a	8.70 (0.33)b	9.42 (0.13)a	12.10 (1.10)b
	**96**	62.33 (2.45)a	63.75 (0.41)a	46.26 (1.19)b	33.66 (1.80)a	42.81 (1.04)b	24.74 (2.09)a	49.61 (0.60)b	31.97 (1.02)a	47.18 (1.47)b	36.72 (1.68)a
	**122**	1.09 (0.05)a	1.11 (0.02)a	4.66 (0.17)b	1.79 (0.07)a	3.54 (0.11)a	8.57 (0.16)b	2.57 (0.05)a	4.34 (0.06)b	3.50 (0.08)a	8.27 (0.08)b
	**126**	/	/	1.82 (0.12)	/	1.34 (0.07)a	2.18 (0.04)b	1.69 (0.04)a	4.41 (0.14)b	/	/
nifH	**153**	/	/	/	6.56 (0.33)	/	3.70 (0.08)	/	5.53 (0.13)	/	2.11 (0.05)
**(2018)**	**188**	16.31 (2.40)a	16.03 (0.65)a	14.52 (0.54)b	9.39 (0.35)a	18.71 (0.82)b	13.33 (1.08)a	17.83 (0.27)b	10.11 (0.41)a	17.64 (1.22)b	13.47 (0.47)a
	**237**	/	/	3.72 (0.06)b	2.16 (0.07)a	3.04 (0.15)a	4.73 (0.11)b	2.51 (0.08)a	3.57 (0.04)b	3.09 (0.04)a	4.40 (0.50)b
	**241**	/	/	/	11.66 (0.61)	1.49 (0.07)a	6.82 (0.61)b	2.27 (0.08)a	8.90 (0.14)b	2.65 (0.14)a	4.60 (0.13)b
	**268**	4.46 (0.19)a	4.59 (0.27)a	12.26 (0.40)a	17.92 (0.69)b	9.93 (0.16)a	14.02 (1.45)b	7.68 (0.13)a	9.81 (0.74)b	7.61 (0.24)a	10.13 (0.63)b
	**450**	/	/	/	/	/	/	1.23 (0.03)a	3.75 (0.19)b	/	/
	**53**	4.77 (0.33)a	4.94 (0.21)a	4.97 (0.62)b	2.80 (0.16)a	2.63 (0.11)a	4.69 (0.19)b	3.43 (0.38)a	4.72 (0.36)b	3.66 (0.15)a	4.22 (0.14)b
	**71**	/	/	1.86 (0.04)	/	1.51 (0.04)a	2.76 (0.15)b	1.14 (0.04)a	1.54 (0.04)b	1.43 (0.05)a	1.87 (0.09)b
	**82**	/	/	/	/	/	1.07 (0.05)	/	2.24 (0.14)	/	1.06 (0.02)
	**137**	/	/	/	/	/	/	/	/	/	2.16 (0.04)
	**329**	14.53 (0.57)a	13.66 (1.43)a	16.49 (0.65)a	22.66 (1.44)b	17.35 (1.50)a	27.76 (2.19)b	23.87 (0.69)a	27.66 (0.37)b	18.39 (1.11)a	22.54 (0.70)b
arch-amoA	**421**	18.08 (1.35)a	17.76 (1.14)a	25.47 (2.26)b	18.18 (1.89)a	23.68 (1.19)b	17.52 (1.83)a	19.73 (0.23)b	14.92 (0.95)a	17.85 (0.45)b	13.56 (0.90)a
**(2017)**	**435**	/	/	1.26 (0.04)	/	1.05 (0.03)a	2.08 (0.04)b	1.38 (0.04)a	2.10 (0.05)b	1.24 (0.02)a	3.76 (0.45)b
	**443**	53.82 (2.47)a	54.63 (2.70)a	31.12 (2.89)a	38.65 (2.14)b	38.79 (2.10)b	26.57 (2.40)a	32.25 (1.46)b	23.91 (1.71)a	40.04 (1.58)b	30.12 (1.93)a
	**555**	/	/	10.01 (0.23)b	7.66 (0.15)a	6.15 (0.27)a	8.59 (0.26)b	9.08 (0.39)a	9.82 (0.20)b	7.50 (0.61)a	8.77 (0.29)b
	**587**	/	/	/	/	/	/	/	3.81 (0.33)	1.72 (0.06)a	3.15 (0.14)b
	**53**	4.59 (0.41)a	4.46 (0.38)a	5.12 (0.40)b	3.49 (0.38)a	3.02 (0.44)a	5.61 (0.34)b	3.08 (0.16)a	4.98 (0.33)b	3.71 (0.24)a	4.72 (0.12)b
arch-amoA	**71**	/	/	2.18 (0.33)	/	1.90 (0.22)a	3.17 (0.31)b	1.04 (0.02)a	1.58 (0.04)b	1.35 (0.02)a	2.53 (0.25)b
**(2018)**	**82**	/	/	/	/	/	1.64 (0.24)	/	1.77 (0.14)	/	1.06 (0.05)
	**137**	/	/	/	/	/	/	/	/	/	2.05 (0.18)

	**329**	14.59 (0.63)a	15.23 (0.84)a	16.40 (0.60)a	20.71 (0.55)b	16.52 (0.78)a	24.97 (1.33)b	21.77 (1.56)a	30.34 (2.99)b	18.84 (0.13)a	21.34 (1.35)b
	**421**	19.02 (0.13)a	18.17 (1.08)a	25.30 (0.81)b	20.85 (0.30)a	25.37 (1.09)b	14.73 (0.17)a	16.70 (0.42)b	13.34 (0.80)a	17.38 (0.44)b	13.76 (0.34)a
	**435**	/	/	1.07 (0.08)	/	1.08 (0.06)a	2.18 (0.21)b	1.12 (0.03)a	3.22 (0.22)b	2.12 (0.06)a	3.09 (0.04)b
	**443**	53.04 (1.31)a	54.69 (1.37)a	30.45 (1.91)a	38.39 (2.14)b	35.72 (1.49)b	28.45 (1.96)a	38.93 (1.28)b	24.03 (0.89)a	38.11 (2.26)b	27.25 (2.41)a
	**555**	/	/	9.62 (0.50)b	7.44 (0.17)a	7.30 (0.39)a	9.96 (0.33)b	7.81 (0.10)a	8.64 (0.09)b	8.31 (0.01)a	9.55 (0.38)b
	**587**	/	/	/	/	/	/	/	3.85 (0.06)	2.00 (0.08)a	4.54 (0.38)b
	**65**	5.84 (0.12)a	6.21 (0.38)a	5.33 (0.12)b	4.78 (0.24)a	2.61 (0.33)a	5.86 (0.03)b	2.47 (0.32)a	6.93 (0.39)b	3.55 (0.12)a	8.17 (0.17)b
	**78**	/	/	/	/	/	/	/	1.19 (0.03)	/	1.58 (0.07)
	**109**	15.60 (1.57)a	16.63 (1.23)a	17.70 (0.46)a	27.72 (2.29)b	15.57 (0.60)a	25.46 (2.94)b	18.85 (0.20)a	25.58 (1.45)b	17.84 (1.64)a	29.29 (1.48)b
amoA	**117**	/	/	/	/	/	1.14 (0.07)	/	3.53 (0.13)	/	4.64 (0.25)
**(2017)**	**208**	/	/	1.29 (0.04)	/	/	/	1.12 (0.04)a	1.26 (0.06)b	1.09 (0.04)a	1.60 (0.07)b
	**270**	1.27 (0.06)a	1.35 (0.09)a	4.10 (0.93)b	2.24 (0.02)a	1.61 (0.09)a	6.06 (0.79)b	1.98 (0.04)a	4.44 (0.06)b	2.48 (0.03)a	3.27 (0.04)b
	**280**	67.92 (1.49)a	66.47 (1.18)a	59.46 (0.45)a	55.70 (3.03)a	69.27 (0.97)b	48.07 (2.40)a	64.33 (0.73)b	43.09 (1.97)a	63.73 (2.32)b	37.51 (0.77)a
	**488**	/	/	3.12 (0.17)	/	2.56 (0.11)a	4.33 (0.04)b	2.68 (0.11)a	5.59 (0.08)b	3.74 (0.31)a	5.47 (0.14)b
	**65**	6.33 (0.40)a	6.01 (0.60)a	6.26 (0.20)b	5.42 (0.29)a	3.40 (0.14)a	7.07 (0.77)b	4.50 (0.38)a	6.83 (0.22)b	4.48 (0.09)a	6.85 (0.71)b
	**78**	/	/	/	/	/	/	/	1.08 (0.03)	/	1.24 (0.04)
	**109**	16.20 (0.39)a	16.45 (1.21)a	17.77 (1.22)a	27.39 (1.95)b	17.01 (0.80)a	25.10 (2.73)b	15.25 (1.50)a	22.91 (1.93)b	17.31 (1.55)a	32.00 (1.77)b
	**117**	/	/	/	/	/	1.42 (0.09)	/	4.58 (0.74)	/	2.97 (0.06)
amoA	**208**	/	/	1.65 (0.15)	/	/	/	2.28 (0.04)a	2.92 (0.14)b	1.48 (0.07)a	2.26 (0.07)b
**(2018)**	**270**	1.41 (0.06)a	1.50 (0.07)a	3.45 (0.10)b	2.53 (0.10)a	2.36 (0.23)a	7.85 (0.41)b	2.18 (0.19)a	3.31 (0.07)b	2.18 (0.08)a	2.87 (0.05)b
	**280**	67.91 (0.69)a	66.69 (1.98)a	57.74 (1.69)a	55.85 (2.85)a	64.02 (1.96)b	43.19 (1.81)a	64.09 (0.97)b	42.76 (3.18)a	62.29 (1.63)b	37.79 (1.00)a
	**488**	/	/	4.58 (0.40)	/	4.33 (0.50)a	5.96 (0.48)b	1.83 (0.18)a	7.03 (0.21)b	3.87 (0.07)a	5.68 (0.07)b

Mean values and SDs of three biological replicates are presented, with each biological replicate representing a pooled sample from at least six individual rhizosphere soils. Values in the same row followed by different letters (a, b) differed significantly between different treatments (PL- and PL+ treatments) at the same sampling stage, and the same letters indicate no significant difference (Student's *t*-test; $p < 0.05$). PL-, noncolonized peanuts; PL+, *P. liquidambaris* colonized peanuts. PR, presowing stage; SS, seedling stage; FS, flowering stage; PS, podding stage; MS, maturing stage. 2017 and 2018, consecutive 2 years field plot experiment.

Table S5 The partial sequence identities of obtained T-RFs from rhizosphere soil diazotrophs, AOA and AOB community clone libraries (field experiment 2 design)

Items	T-RFs (bp)	GenBank accession number	Closest match from GenBank	Coverage/ID (100%)	Number of clones
	**58**	KY311552.1	Uncultured bacterium	78/94	8
	**96**	KC773978.1	Uncultured bacterium	100/100	18
		HQ190136.1	Uncultured bacterium	100/97	5
		KC773961.1	Uncultured bacterium	100/99	12
		KP135636.1	Uncultured bacterium	100/99	2
		KF847352.1	Uncultured bacterium	100/100	8
	**153**	AB079617.1	*Bradyrhizobium* sp.	100/98	6
		KF113072.1	*Bradyrhizobium* sp.	100/97	8
**Diazotrophs**	**188**	KM885202.1	Uncultured bacterium	100/95	15
		KM885208.1	Uncultured bacterium	100/92	10
		AY601051.1	Uncultured bacterium	100/90	3
	**241**	LC107554.1	*Bradyrhizobium japonicum*	100/97	17
		JF789586.1	*Bradyrhizobium* sp.	100/99	8
	**268**	FJ514065.1	*Bradyrhizobium yuanmingense*	98/99	12
		KP219175.1	*Bradyrhizobium sp.*	100/97	6
		GU433561.1	*Bradyrhizobium liaoningense*	99/99	10
	**450**	KC773977.1	Uncultured bacterium	100/100	2
	**53**	KF018534.1	Uncultured archaeon	100/100	5
		JQ014635.1	Uncultured *crenarchaeote*	100/98	2
	**82**	KM402145.1	Uncultured archaeon	100/97	2
		JQ698547.1	Uncultured *crenarchaeote*	100/99	1
		KT152987.1	Uncultured archaeon	99/99	6
	**137**	KP783259.1	Uncultured archaeon	100/100	5
	**329**	KR856811.1	Uncultured archaeon	100/96	12
		KP197319.1	Uncultured archaeon	100/98	3
**AOA**		KR856806.1	Uncultured archaeon	100/97	22
	**421**	HM803767.1	Uncultured *crenarchaeote*	99/99	19
		KT275118.1	Uncultured archaeon	100/100	4
	**443**	MG659085.1	Uncultured archaeon	99/99	5
		KY308636.1	Uncultured *thaumarchaeote*	100/100	16
		KP069264.1	Uncultured archaeon	99/99	8
		MF323899.1	Uncultured archaeon	99/99	32
	**555**	KM030762.1	Uncultured *thaumarchaeote*	100/99	6
	**587**	KC773895.1	Uncultured *crenarchaeote*	100/100	1
	**109**	KR856612.1	Uncultured bacterium	100/99	34
		KU165439.1	Uncultured bacterium	100/97	9

		KJ711573.1	Uncultured bacterium	100/99	18
**AOB**	**208**	KM460415.1	Uncultured bacterium	100/100	6
	**280**	HM113503.1	Uncultured bacterium	100/100	8
		KJ717179.1	Uncultured bacterium	99/99	21
		MF323755.1	Uncultured bacterium	100/98	46
		KX501418.1	Uncultured bacterium	100/99	7

Table S6 Shannon index (*H*) and Evenness index (*E*) of diazotrophs, AOA and AOB communities calculated from T-RFLP data (field experiment 2 design)

Items			PR		SS		FS		PS		MS	
			H	*E*	*H*	*E*	*H*	*E*	*H*	*E*	*H*	*E*
**Diazotrophs**	**2017**	**PL-**	1.04 (0.02)a	0.64 (0.01)a	1.53 (0.03)a	0.78 (0.02)a	1.49 (0.02)a	0.72 (0.01)a	1.40 (0.02)a	0.68 (0.01)a	1.40 (0.02)a	0.67 (0.01)a
		**PL+**	1.05 (0.02)a	0.65 (0.01)a	1.83 (0.02)b	0.88 (0.01)b	1.98 (0.02)b	0.86 (0.01)b	2.12 (0.00)b	0.92 (0.00)b	1.81 (0.01)b	0.87 (0.01)b
	**2018**	**PL-**	0.97 (0.03)a	0.60 (0.02)a	1.49 (0.01)a	0.76 (0.01)a	1.54 (0.02)a	0.74 (0.01)a	1.45 (0.01)a	0.90 (0.01)a	1.44 (0.02)a	0.69 (0.01)a
		**PL+**	0.96 (0.01)a	0.60 (0.01)a	1.76 (0.02)b	0.84 (0.01)b	1.99 (0.02)b	0.91 (0.01)b	2.02 (0.01)b	0.94 (0.01)b	1.76 (0.02)b	0.84 (0.01)b
**AOA**	**2017**	**PL-**	1.08 (0.03)a	0.78 (0.02)a	1.57 (0.02)b	0.81 (0.01)a	1.43 (0.01)a	0.74 (0.01)a	1.52 (0.01)a	0.85 (0.01)b	1.53 (0.02)a	0.74 (0.01)a
		**PL+**	1.07 (0.04)a	0.77 (0.03)a	1.35 (0.02)a	0.84 (0.01)b	1.66 (0.01)b	0.80 (0.01)b	1.79 (0.01)b	0.81 (0.01)a	1.83 (0.02)b	0.79 (0.01)b
	**2018**	**PL-**	1.09 (0.02)a	0.78 (0.01)a	1.58 (0.02)b	0.81 (0.01)a	1.48 (0.01)a	0.76 (0.01)a	1.45 (0.01)a	0.74 (0.01)a	1.58 (0.02)a	0.76 (0.01)a
		**PL+**	1.07 (0.02)a	0.77 (0.01)a	1.37 (0.02)a	0.85 (0.01)b	1.71 (0.01)b	0.82 (0.01)b	1.77 (0.02)b	0.81 (0.01)b	1.89 (0.02)b	0.82 (0.01)b
**AOB**	**2017**	**PL-**	0.75 (0.02)a	0.54 (0.02)a	1.08 (0.02)b	0.60 (0.01)a	0.78 (0.02)a	0.49 (0.01)a	0.91 (0.01)a	0.51 (0.01)a	0.98 (0.04)a	0.55 (0.02)a
		**PL+**	0.78 (0.02)a	0.57 (0.01)a	0.91 (0.02)a	0.65 (0.01)b	1.25 (0.01)b	0.70 (0.01)b	1.46 (0.02)b	0.70 (0.01)b	1.52 (0.01)b	0.73 (0.00)b
	**2018**	**PL-**	0.78 (0.02)a	0.56 (0.01)a	1.14 (0.03)b	0.63 (0.02)a	0.92 (0.04)a	0.57 (0.02)a	0.95 (0.02)a	0.53 (0.01)a	1.01 (0.02)a	0.57 (0.01)a
		**PL+**	0.78 (0.03)a	0.56 (0.03)a	0.93 (0.01)a	0.67 (0.01)b	1.36 (0.01)b	0.76 (0.01)b	1.52 (0.03)b	0.73 (0.02)b	1.47 (0.01)b	0.71 (0.01)b

Mean values and SDs of three biological replicates are presented, with each biological replicate representing a pooled sample from at least six individual rhizosphere soils. Values in the same column followed by different letters (a, b) differed significantly between different treatments (PL- and PL+ treatments) at the same sampling stage, and the same letters indicate no significant difference (Student's *t*-test; $p < 0.05$). PL-, noncolonized peanuts; PL+, *P. liquidambaris* colonized peanuts. PR, presowing stage; SS, seedling stage; FS, flowering stage; PS, podding stage; MS, maturing stage. 2017 and 2018, consecutive 2 years field plot experiment.

Table S7 Soil properties in rhizosphere during the whole growth period of peanuts (field experiment 1 design)

Physicochemical Properties		pH	STN (g kg $^{-1}$)	STP (g kg $^{-1}$)	STK (g kg $^{-1}$)	TOC (g kg $^{-1}$)	DOC (mg kg $^{-1}$)	DON (mg kg $^{-1}$)	AP (mg kg $^{-1}$)	AK (mg kg $^{-1}$)
**PR**	**PL-**	5.41 (0.19)a	0.64 (0.04)a	0.46 (0.03)a	12.65 (0.68)a	6.63 (0.32)a	38.25 (3.08)a	6.39 (0.23)a	12.66 (0.90)a	106.04 (6.86)a
	**PL+**	5.34 (0.11)a	0.62 (0.04)a	0.47 (0.02)a	12.52 (0.36)a	6.54 (0.36)a	38.31 (2.43)a	6.30 (0.20)a	12.64 (0.58)a	103.66 (7.23)a
**SS**	**PL-**	5.34 (0.17)a	0.69 (0.03)a	0.52 (0.03)a	13.29 (0.53)a	7.29 (0.21)a	42.98 (2.75)a	6.93 (0.26)a	20.48 (0.93)a	146.65 (6.61)a
	**PL+**	5.29 (0.08)a	0.68 (0.03)a	0.54 (0.02)a	13.30 (0.41)a	7.99 (0.32)b	50.60 (2.69)b	7.27 (0.15)a	25.87 (1.55)b	152.49 (5.29)a

FS	PL-	5.37 (0.12)a	0.64 (0.03)a	0.49 (0.04)a	12.88 (0.24)a	7.96 (0.34)a	52.24 (2.66)a	8.60 (0.34)a	24.74 (2.19)a	166.07 (7.14)a
	PL+	5.29 (0.14)a	0.67 (0.05)a	0.51 (0.04)a	12.95 (0.34)a	9.04 (0.35)b	61.98 (3.38)b	11.03 (0.88)b	32.63 (2.00)b	194.24 (6.60)b
PS	PL-	5.27 (0.11)a	0.60 (0.02)a	0.43 (0.03)a	12.73 (0.24)a	7.46 (0.33)a	48.71 (3.97)a	8.69 (0.49)a	18.53 (1.05)a	152.16 (3.50)a
	PL+	5.30 (0.08)a	0.62 (0.04)a	0.45 (0.03)a	12.51 (0.62)a	8.29 (0.25)b	58.77 (3.69)b	10.73 (0.72)b	26.56 (1.82)b	169.28 (8.79)b
MS	PL-	5.32 (0.14)a	0.58 (0.04)a	0.40 (0.02)a	12.28 (0.25)a	7.11 (0.30)a	49.72 (3.44)a	8.06 (0.25)a	17.16 (1.04)a	121.03 (4.45)a
	PL+	5.31 (0.11)a	0.60 (0.05)a	0.41 (0.03)a	12.18 (0.30)a	7.89 (0.26)b	58.23 (2.78)b	9.87 (0.64)b	22.18 (1.19)b	138.90 (6.46)b

Mean values and SDs of three biological replicates are presented, with each biological replicate representing a pooled sample from at least six individual rhizosphere soils. Values in the same column followed by different letters (a, b) differed significantly between different treatments (PL- and PL+ treatments) at the same sampling stage, and the same letters indicate no significant difference (Student's *t*-test; $p < 0.05$). PL-, noncolonized peanuts; PL+, *P. liquidambaris* colonized peanuts. PR, presowing stage; SS, seedling stage; FS, flowering stage; PS, podding stage; MS, maturing stage. STN, soil total nitrogen; STP, soil total phosphorus; STK, soil total potassium; TOC, total organic carbon; DOC, dissolved organic carbon; DON, dissolved organic nitrogen; AP, available phosphorus; AK, available potassium. 2017 and 2018, consecutive 2 years field plot experiment.

Table S8 Soil properties in rhizosphere during the whole growth period of peanuts (field experiment 2 design)

Item			pH	STN (g kg-1)	STP (g kg-1)	STK (g kg-1)	TOC (g kg-1)	DOC (mg kg-1)	DON (mg kg-1)	AP (mg kg-1)	AK (mg kg -1)
2017	PR	PL-	7.20 (0.04)a	0.83 (0.05)a	0.62 (0.02)a	9.13 (0.10)a	8.05 (0.11)a	44.82 (1.51)a	5.68 (0.17)a	16.25 (0.54)a	115.02 (7.64)a
		PL+	7.12 (0.07)a	0.85 (0.04)a	0.62 (0.04)a	9.14 (0.08)a	8.06 (0.05)a	43.66 (1.44)a	5.70 (0.10)a	16.44 (0.56)a	116.50 (5.47)a
	SS	PL-	7.17 (0.06)a	0.88 (0.04)a	0.68 (0.03)a	9.63 (0.24)a	8.63 (0.12)a	48.97 (1.88)a	6.03 (0.09)a	27.06 (0.90)a	142.24 (3.94)a
		PL+	7.20 (0.06)a	0.89 (0.04)a	0.66 (0.03)a	9.68 (0.38)a	9.13 (0.08)b	59.25 (1.96)b	6.53 (0.14)b	30.56 (1.08)b	148.36 (3.89)a
	FS	PL-	7.13 (0.09)a	0.85 (0.03)a	0.64 (0.03)a	9.35 (0.31)a	8.95 (0.12)a	54.41 (1.96)a	7.83 (0.12)a	29.86 (1.16)a	153.84 (5.03)a
		PL+	7.16 (0.04)a	0.84 (0.04)a	0.65 (0.04)a	9.38 (0.22)a	9.97 (0.20)b	67.80 (2.56)b	9.34 (0.12)b	36.28 (1.67)b	176.03 (5.77)b
	PS	PL-	7.10 (0.05)a	0.82 (0.03)a	0.61 (0.05)a	9.09 (0.28)a	8.45 (0.09)a	51.69 (1.58)a	8.02 (0.12)a	21.95 (1.20)a	145.43 (5.02)a
		PL+	7.15 (0.04)a	0.81 (0.03)a	0.62 (0.04)a	9.18 (0.10)a	9.07 (0.05)b	60.16 (1.25)b	9.17 (0.08)b	28.34 (1.38)b	162.55 (3.69)b

	MS	PL-	7.18 (0.06)a	0.82 (0.05)a	0.62 (0.03)a	9.12 (0.17)a	8.16 (0.06)a	46.56 (1.59)a	6.41 (0.07)a	19.67 (1.50)a	123.10 (2.71)a
		PL+	7.15 (0.06)a	0.84 (0.02)a	0.64 (0.02)a	9.15 (0.09)a	8.52 (0.14)b	56.18 (1.52)b	7.95 (0.12)b	24.29 (1.22)b	142.78 (6.05)b
2018	PR	PL-	7.15 (0.07)a	0.84 (0.03)a	0.60 (0.02)a	8.92 (0.14)a	8.14 (0.09)a	46.80 (1.26)a	5.89 (0.08)a	15.40 (0.69)a	108.23 (4.51)a
		PL+	7.12 (0.09)a	0.85 (0.02)a	0.61 (0.03)a	8.84 (0.09)a	8.16 (0.09)a	47.49 (1.88)a	5.90 (0.07)a	15.39 (0.86)a	105.07 (2.86)a
	SS	PL-	7.12 (0.10)a	0.90 (0.02)a	0.65 (0.03)a	9.15 (0.07)a	8.56 (0.27)a	54.01 (1.65)a	6.53 (0.10)a	25.89 (1.03)a	138.63 (3.59)a
		PL+	7.13 (0.08)a	0.88 (0.05)a	0.64 (0.03)a	9.13 (0.11)a	9.27 (0.15)b	62.35 (1.67)b	6.89 (0.14)b	28.33 (1.04)b	148.72 (4.28)b
	FS	PL-	7.13 (0.10)a	0.86 (0.02)a	0.65 (0.04)a	9.16 (0.11)a	9.05 (0.11)a	58.94 (3.14)a	7.60 (0.12)a	25.69 (1.44)a	143.12 (4.75)a
		PL+	7.08 (0.06)a	0.87 (0.04)a	0.66 (0.03)a	9.19 (0.04)a	10.38 (0.64)b	74.36 (2.81)b	9.84 (0.08)b	34.77 (1.64)b	161.89 (3.89)b
	PS	PL-	7.09 (0.06)a	0.85 (0.04)a	0.61 (0.04)a	8.92 (0.13)a	8.47 (0.13)a	56.31 (1.09)a	8.14 (0.11)a	22.70 (1.10)a	134.36 (5.47)a
		PL+	7.11 (0.08)a	0.84 (0.04)a	0.62 (003)a	8.97 (0.13)a	8.82 (0.08)b	68.38 (2.00)b	9.42 (0.17)b	30.25 (0.71)b	152.11 (3.45)b
	MS	PL-	7.13 (0.08)a	0.84 (0.02)a	0.61 (0.03)a	8.90 (0.09)a	8.20 (0.09)a	52.48 (1.70)a	6.61 (0.11)a	19.05 (1.38)a	114.42 (3.82)a
		PL+	7.14 (0.07)a	0.83 (0.02)a	0.60 (0.04)a	8.96 (0.10)a	8.50 (0.08)b	62.48 (1.57)b	7.88 (0.14)b	24.45 (1.08)b	130.70 (7.09)b

Mean values and SDs of three biological replicates are presented, with each biological replicate representing a pooled sample from at least six individual rhizosphere soils. Values in the same column followed by different letters (a, b) differed significantly between different treatments (PL- and PL+ treatments) at the same sampling stage, and the same letters indicate no significant difference (Student's *t*-test; $p < 0.05$). PL-, noncolonized peanuts; PL+, *P. liquidambaris* colonized peanuts. PR, presowing stage; SS, seedling stage; FS, flowering stage; PS, podding stage; MS, maturing stage. STN, soil total nitrogen; STP, soil total phosphorus; STK, soil total potassium; TOC, total organic carbon; DOC, dissolved organic carbon; DON, dissolved organic nitrogen; AP, available phosphorus; AK, available potassium. 2017 and 2018, consecutive 2 years field plot experiment.

Table S9 Determination of the dynamic changes of root exudate components during the whole growth period of peanuts (field experiment 1 design)

Item		Total C	Total N	Soluble sugar	Amino acids	Organic acids	Phenolics	Flavonoids
SS	PL-	52.24 (3.79)a	10.53 (0.76)a	62.63 (4.40)a	5.15 (0.06)a	44.30 (2.81)a	0.38 (0.03)a	14.00 (0.99)a
	PL+	66.62 (4.05)b	13.61 (1.18)b	85.65 (5.61)b	5.86 (0.12)b	64.55 (4.50)b	0.70 (0.06)b	25.14 (1.75)b
FS	PL-	157.79 (7.07)a	31.14 (2.16)a	188.50 (6.04)a	13.34 (0.59)a	113.82 (6.23)a	0.73 (0.03)a	31.81 (2.44)a

	PL+	196.67 (8.76)b	39.83 (1.60)b	242.00 (6.11)b	15.94 (0.34)b	152.17 (7.35)b	0.96 (0.06)b	46.74 (2.09)b
PS	PL-	229.53 (6.81)a	45.19 (2.82)a	264.70 (6.59)a	16.29 (0.51)a	136.45 (5.08)a	1.26 (0.06)a	54.87 (2.71)a
	PL+	263.62 (6.66)b	53.66 (2.25)b	310.31 (6.90)b	17.96 (0.66)b	164.46 (4.61)b	1.23 (0.05)a	55.44 (4.16)a
MS	PL-	99.19 (5.83)a	23.69 (2.33)a	119.10 (4.91)a	10.65 (0.92)a	86.84 (4.05)a	0.78 (0.04)b	27.31 (2.36)b
	PL+	125.81 (5.21)b	31.44 (1.86)b	139.08 (6.88)b	12.17 (0.55)b	90.40 (6.26)a	0.66 (0.03)a	22.13 (2.27)a

Mean values and SDs of three biological replicates are presented, with each biological replicate representing a pooled sample from at least six individual peanut plants. Values in the same column followed by different letters (a, b) differed significantly between different treatments (PL- and PL+ treatments) at the same sampling stage, and the same letters indicate no significant difference (Student's *t*-test; $p < 0.05$). PL-, noncolonized peanuts; PL+, *P. liquidambaris* colonized peanuts. SS, seedling stage; FS, flowering stage; PS, podding stage; MS, maturing stage. Measure unit: flavonoids compounds, ng plant^{-1}; other components in the peanut root exudates, μg plant^{-1}.

Table S10 Effects of *P. liquidambaris* colonization and coinoculation with native *Bradyrhizobium* on peanut nodulation parameters under the different N fertilizer conditions (field experiment 3 design)

Item	Non		BMI		P		P+BMI	
	Low N	Normal N	Low N	Normal N	Low N	Normal N	Low N	Normal N
**Total nodule weight (mg plnat^{-1})**	32.91 (1.28)b	26.77 (1.56)a	51.74 (1.47)e	48.04 (1.70)d	44.81 (1.89)c	42.47 (1.45)c	75.09 (2.82)g	62.29 (1.56)f
**Nodule number g^{-1} root DW**	42.55 (4.40)d	22.71 (2.39)a	60.00 (4.04)e	37.14 (2.58)c	46.22 (3.81)d	29.96 (3.08)b	63.30 (4.84)e	43.24 (2.91)d
**Nodule DW (mg) g^{-1} root DW**	78.21 (3.18)d	41.73 (2.72)a	107.63 (7.73)f	70.39 (3.60)c	88.39 (5.61)e	56.93 (4.48)b	126.11 (8.04)g	82.34 (3.66)de
**Leghemoglobin content (μmol g^{-1} FW nodule)**	0.27 (0.02)a	0.36 (0.02)c	0.32 (0.02)b	0.45 (0.02)de	0.35 (0.02)bc	0.42 (0.02)d	0.38 (0.02)c	0.46 (0.03)e
**Nitrogenase activity (μmol h^{-1} g^{-1} DW nodule)**	36.09 (2.25)a	43.55 (2.08)bc	52.83 (2.10)d	46.81 (2.00)c	40.80 (2.40)b	58.99 (3.29)e	75.14 (2.84)g	63.37 (2.01)f

Mean values and SDs of three biological replicates are presented, with each biological replicate representing a pooled sample from at least six individual plants. Values in the same row followed by different letters differed significantly among treatments, and the same letters indicate no significant difference (one-way ANOVA with Tukey's test, $p < 0.05$). Non, noninoculated seedlings; BMI, seedlings only inoculated with BMI (bradyrhizobium mixed inoculant); P, seedlings only inoculated with *P. liquidambaris*; P+BMI, seedlings coinoculated with *P. liquidambaris* and BMI; DW, dry weight; Low or Normal N, low or normal N fertilizer.

Figure Legends

Figure S1 *P. liquidambaris* colonization significantly increases nodulation, N_2 fixation and plant biomass of continuous cropping peanuts in field experiment 1. (a-d) determination of nodule number, nodule dry weight and specific nodule parameters at flowering stage; (e-h) symbiosis related gene expressions of *SymRK*, *CCaMK*, *NIN* and *ENOD40* in roots at the nodule initiation stage; (i-k) N accumulation in shoot, root and pod of peanuts during the whole growth stage; (l, m) shoot and root dry weight of peanuts during the whole growth stage. Data and errors are mean ± SD (n=3) and asterisks indicate significant differences between the PL- and PL+ treatments in each growing stage (Student's *t*-test; $p < 0.05$). Each biological replicate representing a pooled sample from at least six individual plants. PL-, noncolonized peanuts; PL+, *P. liquidambaris* colonized peanuts; SS, seedling stage; FS, flowering stage; PS, podding stage; MS, maturing stage; DW, dry weight.

Figure S2 *P. liquidambaris* colonization significantly increases nodulation, N_2 fixation and plant biomass of continuous cropping peanuts in field experiment 2. (a-d) determination of nodule number, nodule dry weight and specific nodule parameters at flowering stage; (e, f) nodule N_2 fixation capacity detection; (g-i) N accumulation in shoot, root and pod of peanuts during the whole growth stage; (j, k) shoot and root dry weight of peanuts during the whole growth stage. Data and errors are mean ± SD (n=3) and asterisks indicate significant differences between the PL- and PL+ treatments in each growing stage (Student's *t*-test; $p < 0.05$). Each biological replicate representing a pooled sample from at least six individual plants. PL-, noncolonized peanuts; PL+, *P. liquidambaris* colonized peanuts; SS, seedling stage; FS, flowering stage; PS, podding stage; MS, maturing stage; DW, dry weight. 2017 and 2018, consecutive 2 years field plot experiment.

Figure S3 *P. liquidambaris* colonization significantly affects the level of available N in rhizosphere. Dynamic changes of NH_4^+-N, NO_3^--N concentrations, and soil PNR in peanuts rhizosphere of field experiment 1 (a-c) and field experiment 2 (d-f) during the whole growth stage. Data and errors are mean ± SD (n=3) and asterisks indicate significant differences between the PL- and PL+ treatments in each growing stage (Student's *t*-test; $p < 0.05$). Each biological replicate representing a pooled sample from at least six individual rhizosphere soils. PL-, noncolonized peanuts; PL+, *P. liquidambaris* colonized peanuts; PR, presowing stage; SS, seedling stage; FS, flowering stage; PS, podding stage; MS, maturing stage; PNR, potential nitrification rate. 2017 and 2018, consecutive 2 years field plot experiment.

Figure S4 Dynamic changes of available N in rhizosphere caused by *P. liquidambaris* colonization are beneficial to peanut-bradyrhizobium symbiosis. Symbiotic interaction of peanut-bradyrhizobium (nodule initiation stage) was analyzed under 0.5 mM NH_4^+-N and NO_3^--N conditions. (a-d) symbiosis related gene expressions of *SymRK*, *CCaMK*, *NIN* and *ENOD40* in peanut roots; (e-j) detection of nodulation signaling molecules in peanut roots. Data and errors are mean ± SD (n=3), and different letters indicate significant differences among treatments (one-way ANOVA

with Tukey's test, $p < 0.05$). Each biological replicate representing a pooled sample from at least six individual plants. FW, fresh weight.

Figure S5 *P. liquidambaris* colonization significantly enhances the symbiosis interaction of peanut-bradyrhizobium in field experiment 2. Symbiosis related gene expressions of *SymRK*, *CCaMK*, *NIN* and *ENOD40* (a-d), and the levels of nodulation signaling molecules (e-j) were analyzed in roots at the nodule initiation stage. Data and errors are mean ± SD (n=3) and asterisks indicate significant differences between the PL- and PL+ treatments (Student's *t*-test; $p < 0.05$). Each biological replicate representing a pooled sample from at least six individual plants. PL-, noncolonized peanuts; PL+, *P. liquidambaris* colonized peanuts; FW, fresh weight; 2017 and 2018, consecutive 2 years field plot experiment.

Figure S6 Effects of inoculation of native *Bradyrhizobium* on nodule parameters, shoot and root N contents of peanuts at the flowering stage. Data and errors are mean ± SD (n=3), and different letters indicate significant differences among treatments (one-way ANOVA with Tukey's test, $p < 0.05$). Each biological replicate representing a pooled sample from at least six individual plants. H_2O, sterile distilled water treatment; B-1, inoculation of *Bradyrhizobium japonicum*; B-2, inoculation of *Bradyrhizobium* sp.; B-3, inoculation of *Bradyrhizobium liaoningense*; DW, dry weight.

Figure S7 Colonization amount of fungal endophyte *P. liquidambaris* in roots of peanuts during the whole growth stage in field experiment 1. Data and errors are mean ± SD (n=3), and different letters indicate significant differences between seedling and flowering stages (Student's *t*-test; $p < 0.05$). Each biological replicate representing a pooled sample from at least six individual plants. SS, seedling stage; FS, flowering stage; PS, podding stage; MS, maturing stage; nd, no detected.

Figure S8 Coinoculation *P. liquidambaris* with native *Bradyrhizobium* enhance peanut-bradyrhizobium symbiosis interaction of continuous cropping peanuts under low N fertilizer conditions. Symbiosis related gene expressions of *SymRK*, *CCaMK*, *NIN* and *ENOD40* (a-d), and the levels of nodulation signaling molecules (e-j) were analyzed in roots at the nodule initiation stage. Data and errors are mean ± SD (n=3), and different letters indicate significant differences among treatments (one-way ANOVA with Tukey's test, $p < 0.05$). Each biological replicate representing a pooled sample from at least six individual plants. Non, noninoculated seedlings; BMI, seedlings only inoculated with BMI (bradyrhizobium mixed inoculant); P, seedlings only inoculated with *P. liquidambaris*; P+BMI, seedlings coinoculated with *P. liquidambaris* and BMI; Low or Normal N, low or normal N fertilizer; FW, fresh weight.

Figure S9 Coinoculation *P. liquidambaris* with native *Bradyrhizobium* improve rhizosphere N transforming microbial abundance and available N level throughout the growth of peanuts under low N fertilizer conditions (field experiment 3). (a-c, g-i) abundances of N transforming microbial communities [*nifH* (diazotrophs), *arch-amoA* (AOA) and *amoA* (AOB) genes] in peanuts rhizosphere at nodule initiation and

mid-late growth stages; (d-f, j-l) dynamic changes of NH_4^+-N, NO_3^--N concentrations, and soil PNR in peanuts rhizosphere at nodule initiation and mid-late growth stages. Data and errors are mean ± SD (n=3), and different letters indicate significant differences among treatments (one-way ANOVA with Tukey's test, $p < 0.05$). Each biological replicate representing a pooled sample from at least six individual rhizosphere soils. Non, noninoculated seedlings; BMI, seedlings only inoculated with BMI (bradyrhizobium mixed inoculant); P, seedlings only inoculated with *P. liquidambaris*; P+BMI, seedlings coinoculated with *P. liquidambaris* and BMI; NIS, nodule initiation stage; M-LS, mid-late stage; PNR, potential nitrification rate. Low or Normal N, low or normal N fertilizer.

Figure S1

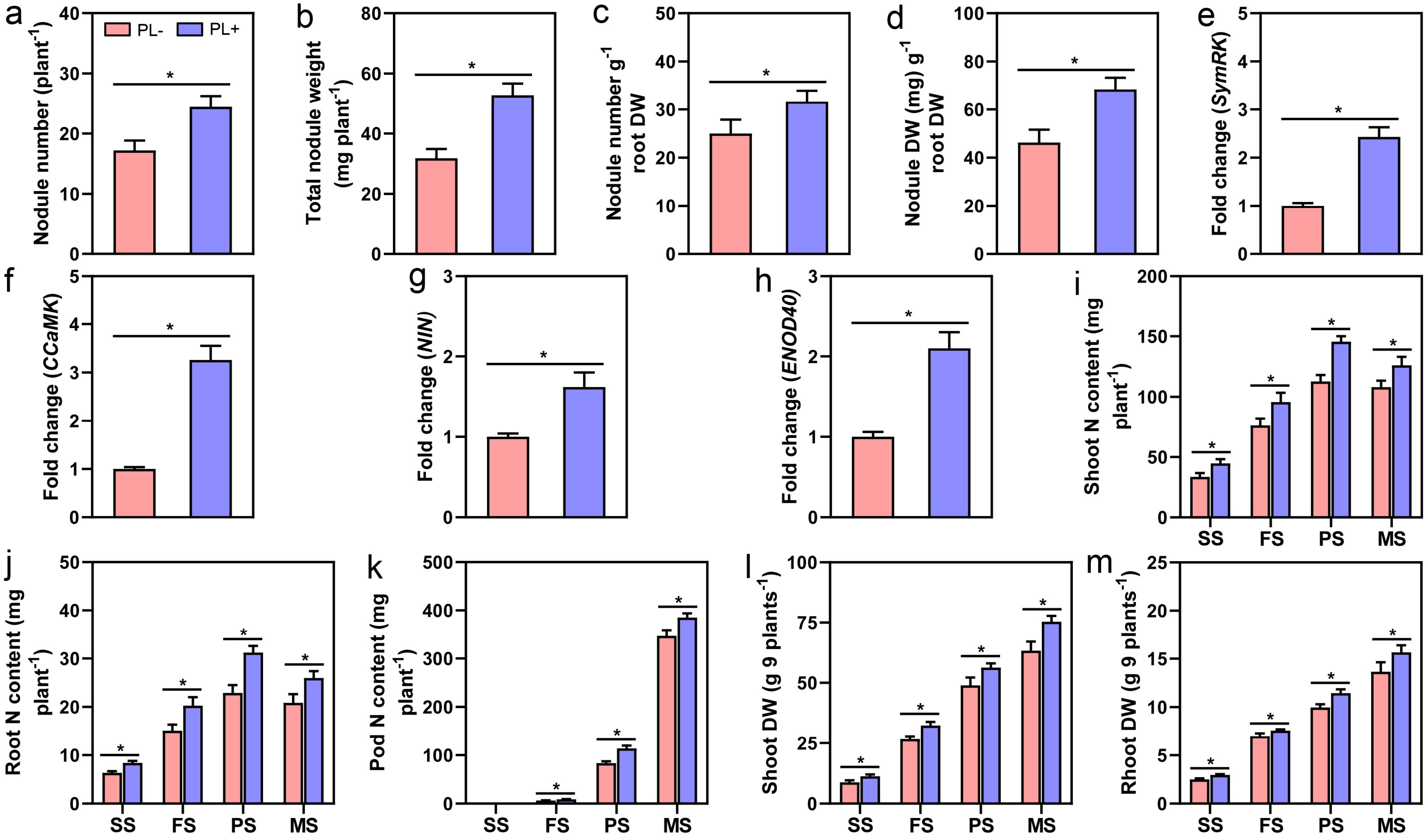

a
b
c
d
e
f
g
h
i
j
k
l
m
PL-
PL+
Nodule number (plant-1)
Total nodule weight (mg plant-1)
Nodule number g-1 root DW
Nodule DW (mg) g-1 root DW
Fold change (SymRK)
Fold change (CCaMK)
Fold change (NIN)
Fold change (ENOD40)
Shoot N content (mg plant-1)
Root N content (mg plant-1)
Pod N content (mg plant-1)
Shoot DW (g 9 plants-1)
Rhoot DW (g 9 plants-1)
SS
FS
PS
MS

Figure S2

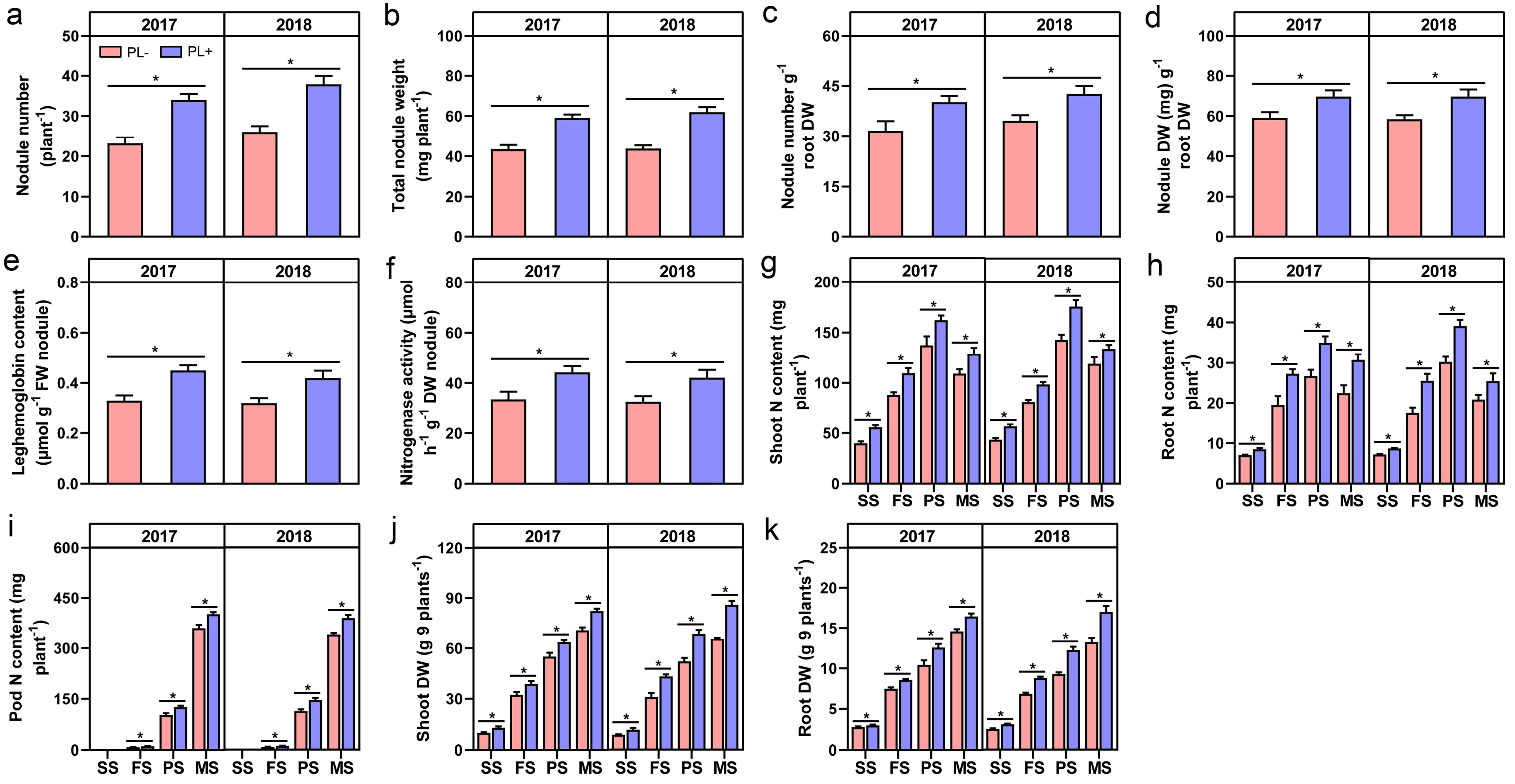
a
b
c
d
e
f
g
h
i
j
k
2017
2018
PL-
PL+
Nodule number (plant-1)
Total nodule weight (mg plant-1)
Nodule number g-1 root DW
Nodule DW (mg) g-1 root DW
Leghemoglobin content (μmol g-1 FW nodule)
Nitrogenase activity (μmol h-1 g-1 DW nodule)
Shoot N content (mg plant-1)
Root N content (mg plant-1)
Pod N content (mg plant-1)
Shoot DW (g 9 plants-1)
Root DW (g 9 plants-1)
SS
FS
PS
MS

Figure S3

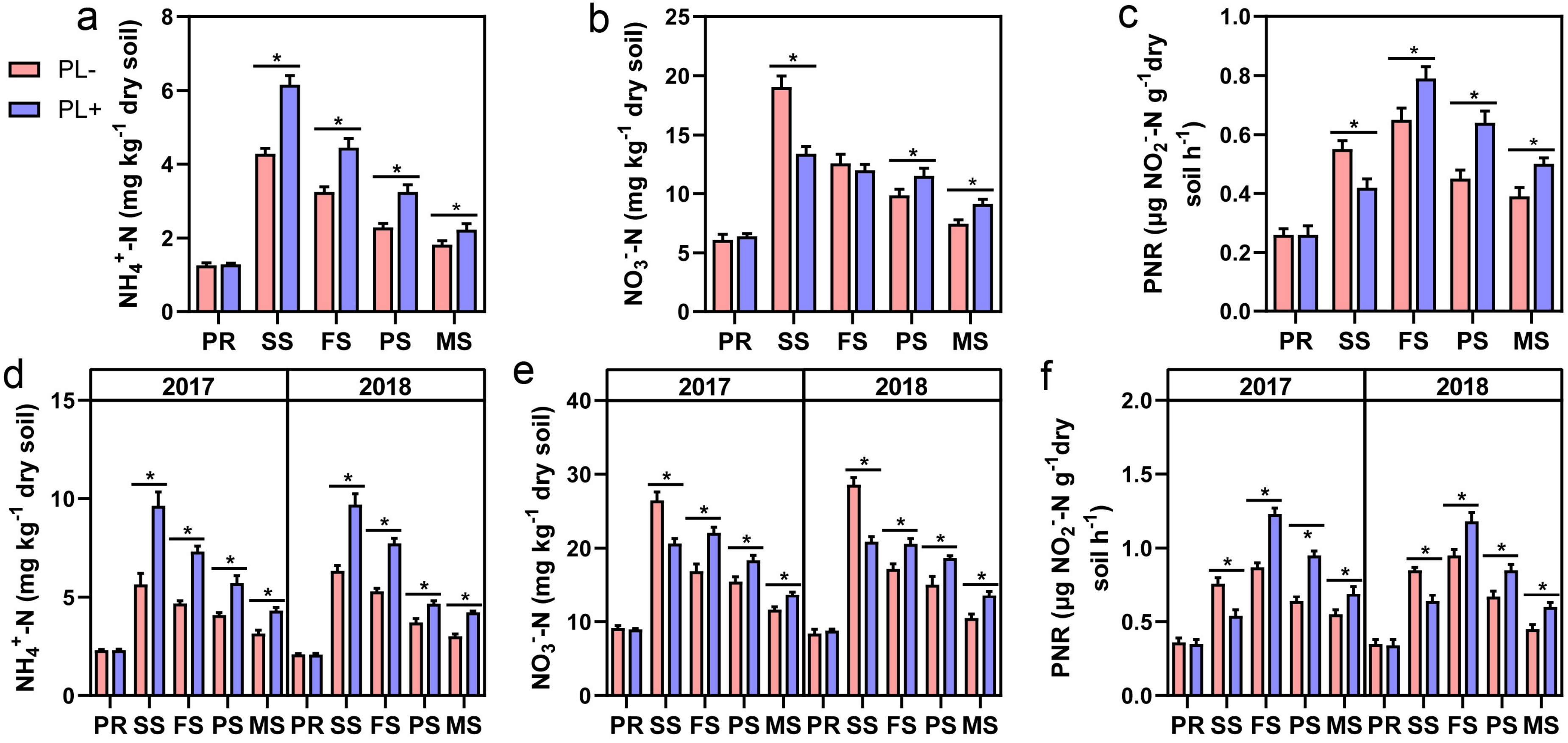
a
b
c
d
e
f
PL-
PL+
NH4+-N (mg kg-1 dry soil)
NO3--N (mg kg-1 dry soil)
PNR (µg NO2--N g-1dry soil h-1)
PR
SS
FS
PS
MS
2017
2018
*

Figure S4

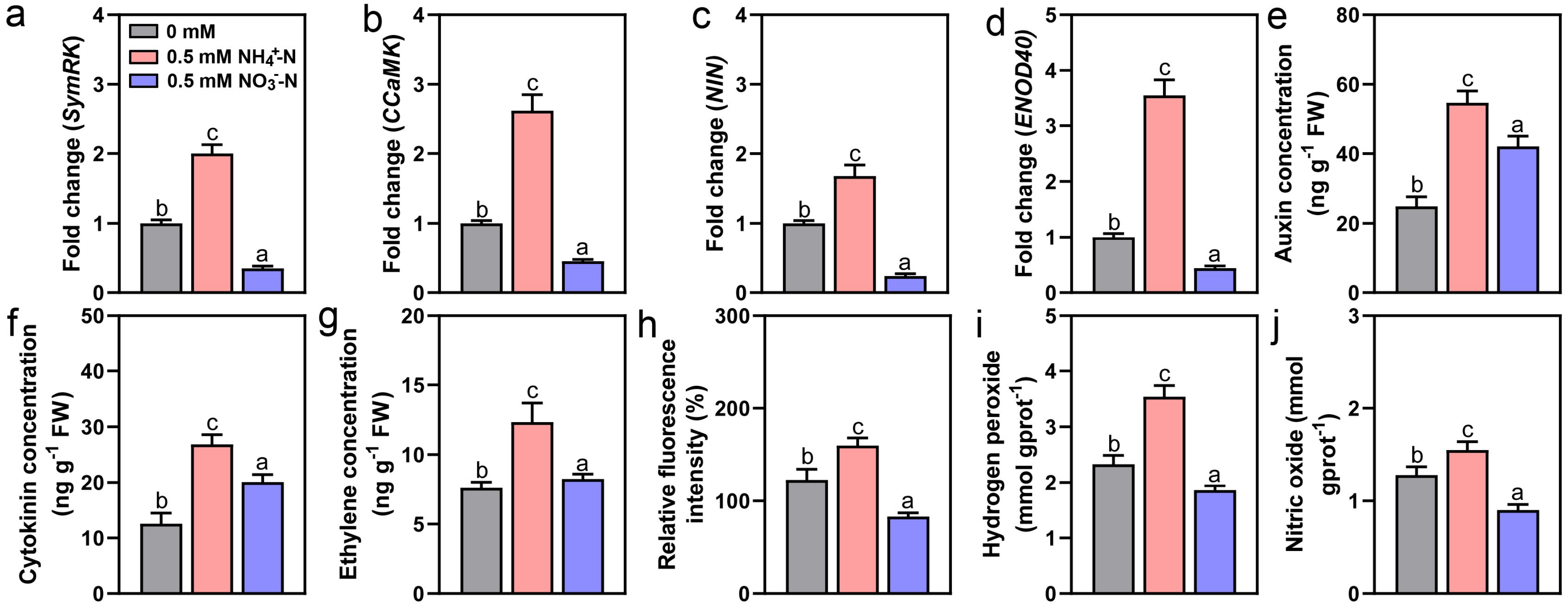

a
b
c
d
e
f
g
h
i
j
0 mM
0.5 mM NH4+-N
0.5 mM NO3−-N
Fold change (SymRK)
Fold change (CCaMK)
Fold change (NIN)
Fold change (ENOD40)
Auxin concentration (ng g-1 FW)
Cytokinin concentration (ng g-1 FW)
Ethylene concentration (ng g-1 FW)
Relative fluorescence intensity (%)
Hydrogen peroxide (mmol gprot-1)
Nitric oxide (mmol gprot-1)

Figure S5

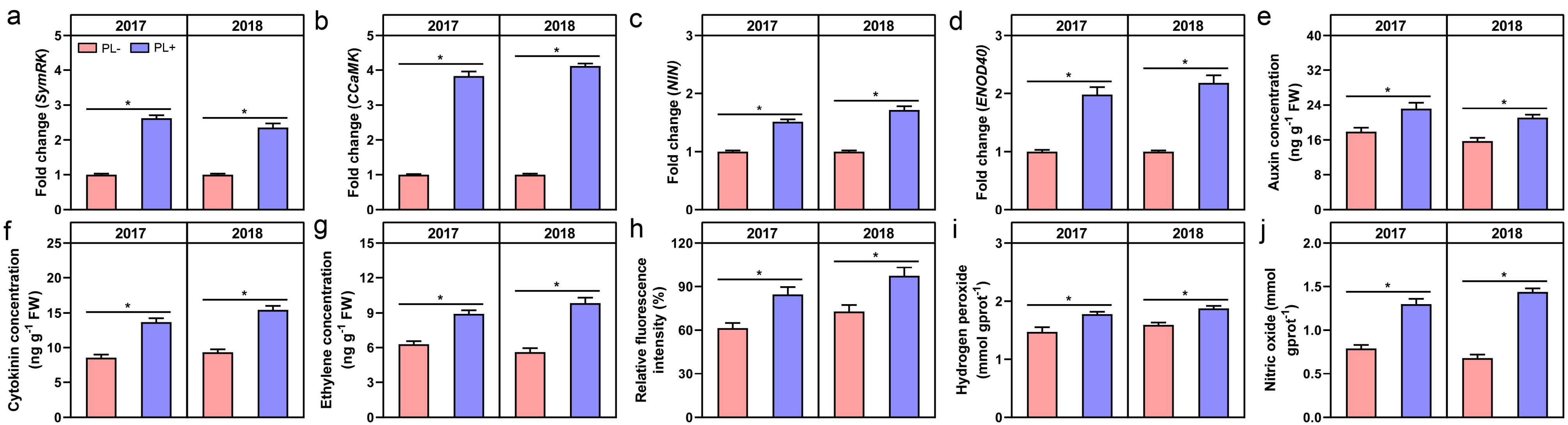
a
b
c
d
e
f
g
h
i
j
2017
2018
PL-
PL+
Fold change (*SymRK*)
Fold change (*CCaMK*)
Fold change (*NIN*)
Fold change (*ENOD40*)
Auxin concentration (ng g^{-1} FW)
Cytokinin concentration (ng g^{-1} FW)
Ethylene concentration (ng g^{-1} FW)
Relative fluorescence intensity (%)
Hydrogen peroxide (mmol gprot^{-1})
Nitric oxide (mmol gprot^{-1})

Figure S6

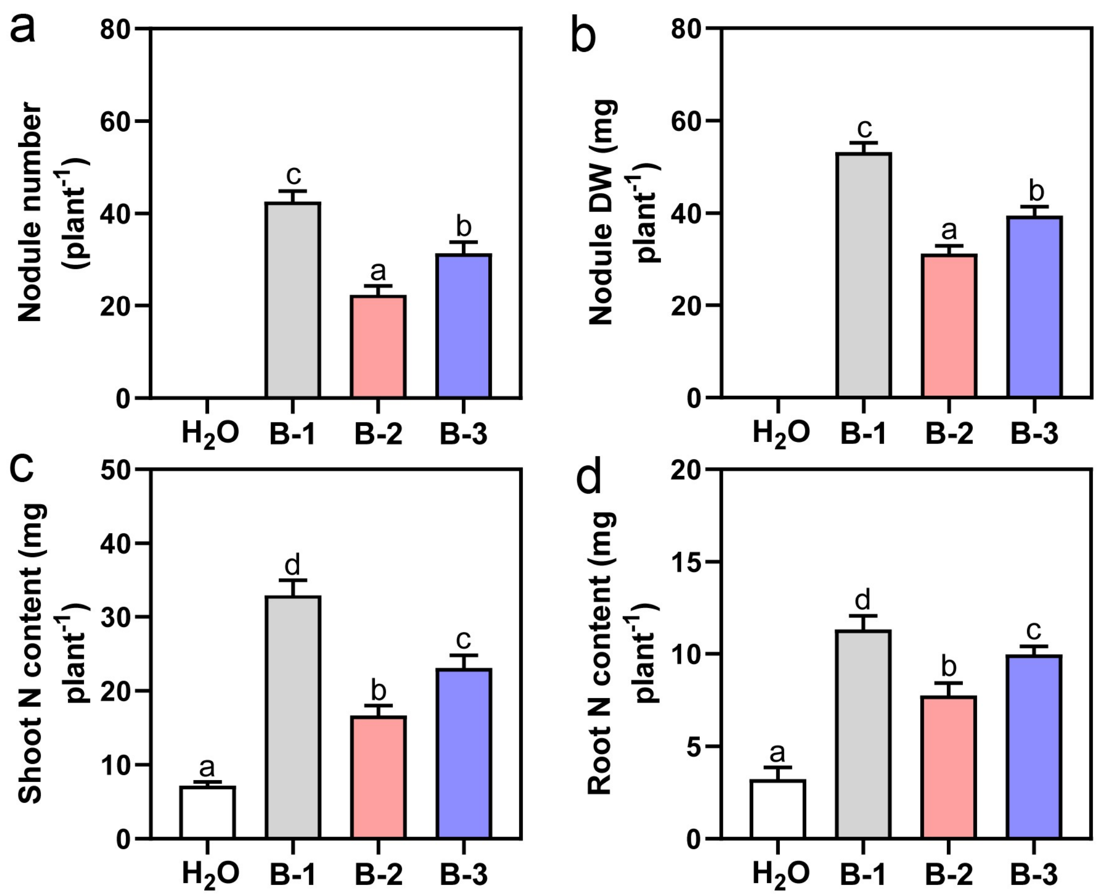

Figure S7

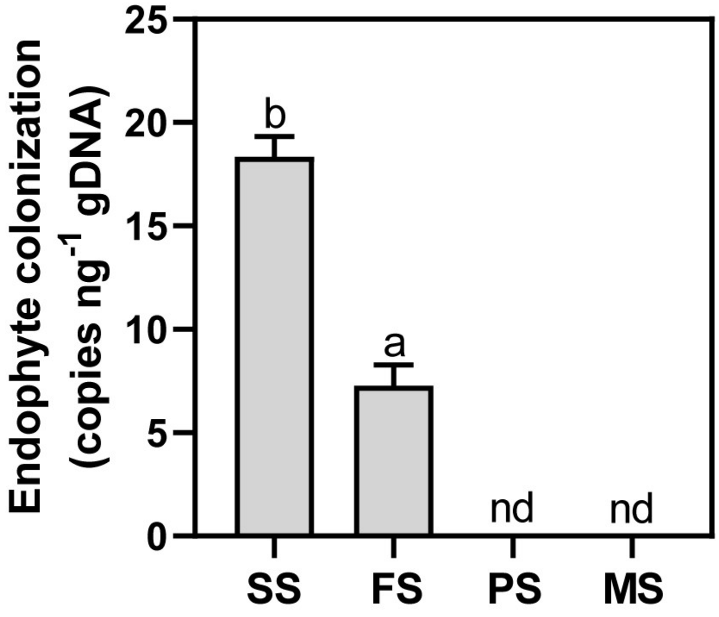

Figure S8

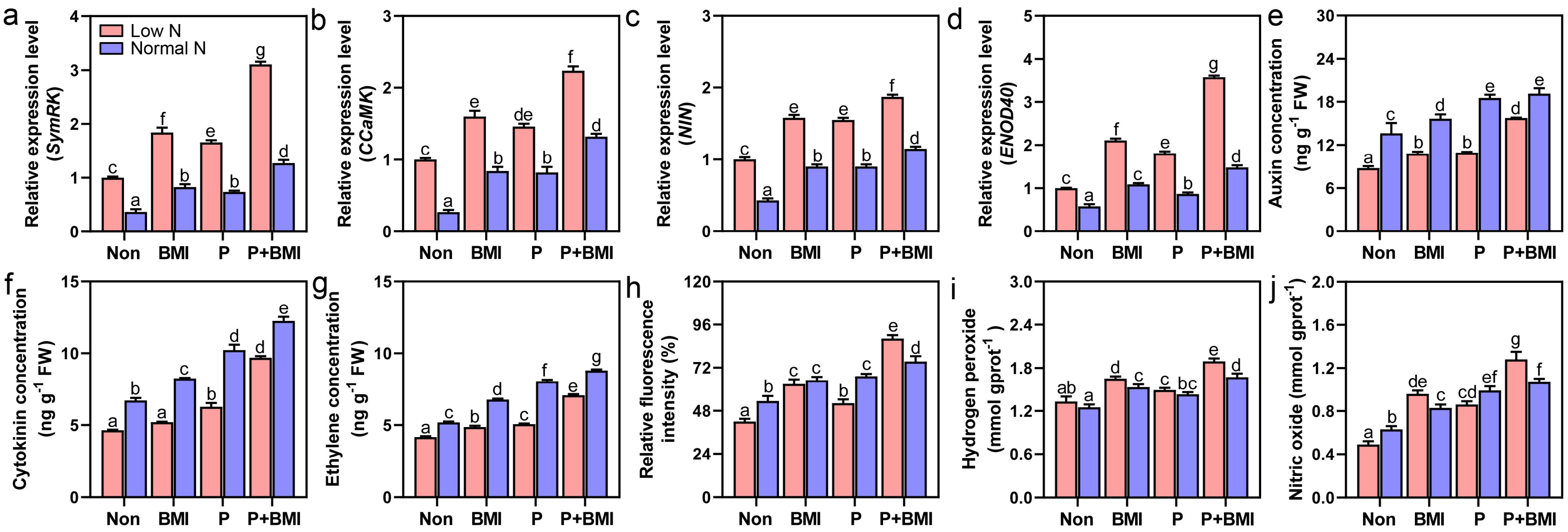
a
b
c
d
e
f
g
h
i
j
Low N
Normal N
Relative expression level (SymRK)
Relative expression level (CCaMK)
Relative expression level (NIN)
Relative expression level (ENOD40)
Auxin concentration (ng g-1 FW)
Cytokinin concentration (ng g-1 FW)
Ethylene concentration (ng g-1 FW)
Relative fluorescence intensity (%)
Hydrogen peroxide (mmol gprot-1)
Nitric oxide (mmol gprot-1)
Non
BMI
P
P+BMI

Figure S9

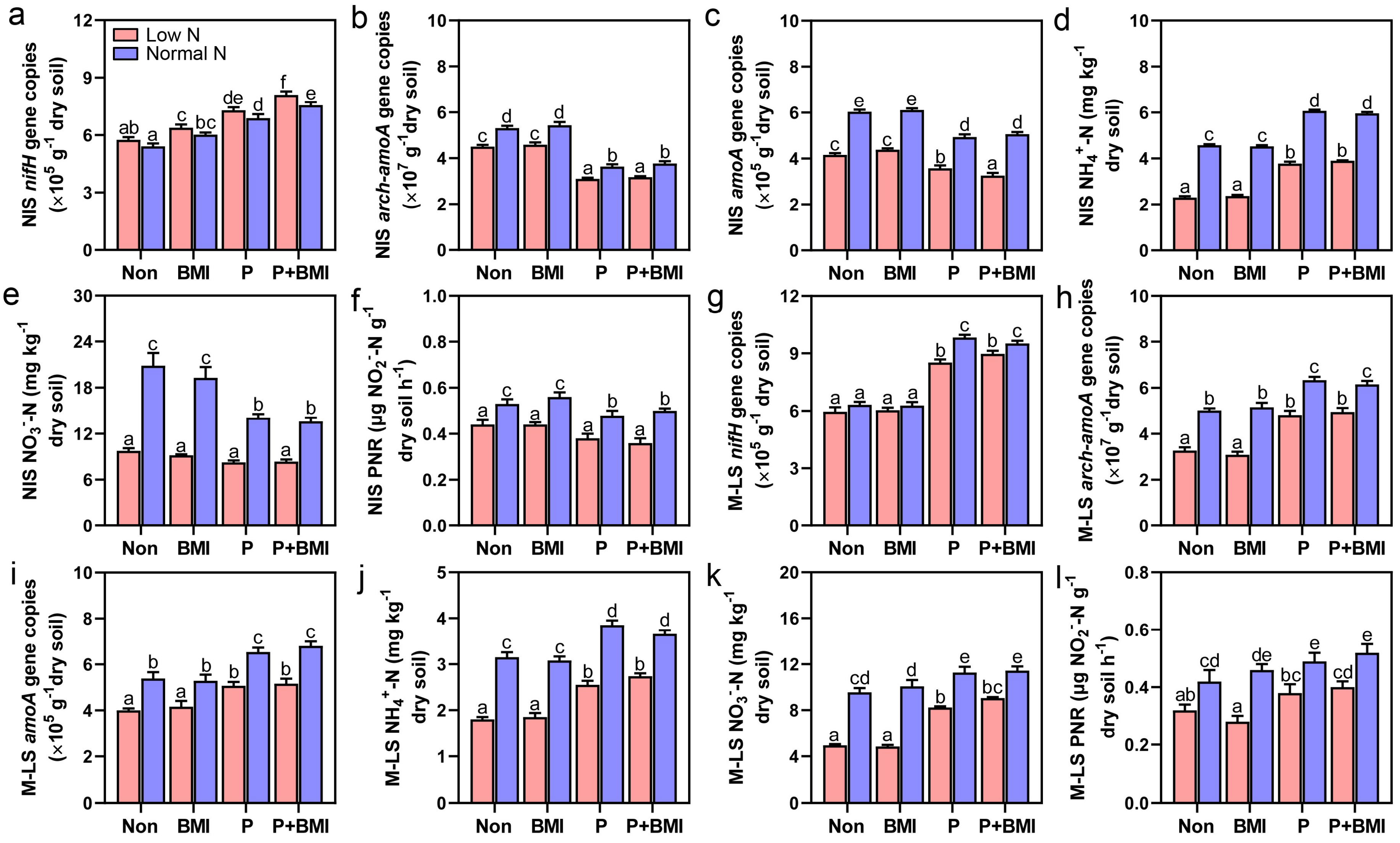
a
Low N
Normal N
NIS nifH gene copies (×10^5 g^-1 dry soil)
Non BMI P P+BMI
b
NIS arch-amoA gene copies (×10^7 g^-1dry soil)
c
NIS amoA gene copies (×10^5 g^-1dry soil)
d
NIS NH4+-N (mg kg^-1 dry soil)
e
NIS NO3−-N (mg kg^-1 dry soil)
f
NIS PNR (μg NO2−-N g^-1 dry soil h^-1)
g
M-LS nifH gene copies (×10^5 g^-1 dry soil)
h
M-LS arch-amoA gene copies (×10^7 g^-1dry soil)
i
M-LS amoA gene copies (×10^5 g^-1dry soil)
j
M-LS NH4+-N (mg kg^-1 dry soil)
k
M-LS NO3−-N (mg kg^-1 dry soil)
l
M-LS PNR (μg NO2−-N g^-1 dry soil h^-1)
